# Metabolomic Investigation of *Citrus latifolia* and the Putative Role of Coumarins in Resistance to Black Spot Disease

**DOI:** 10.3389/fmolb.2022.934401

**Published:** 2022-06-24

**Authors:** Hocelayne Paulino Fernandes, Luis Francisco Salomé-Abarca, Rosana Gonçalves Pereira, Janaína Brandão Seibert, Geraldo José Silva-Junior, Maria Fátima Das Graças Fernandes da Silva, Young Hae Choi

**Affiliations:** ^1^ Natural Products Laboratory, Department of Chemistry, Federal University of São Carlos, São Carlos, Brazil; ^2^ Natural Products Laboratory, Institute of Biology, Leiden University, Leiden, Netherlands; ^3^ Fund for Citrus Protection, Fundecitrus Araraquara, Araraquara, Brazil; ^4^ College of Pharmacy, Kyung Hee University, Seoul, South Korea

**Keywords:** citrus, coumarins, metabolomics, pant defense, pathogen

## Abstract

Citrus black spot (CBS) is a disease caused by the fungus *Phyllosticta citricarpa* that affects citrus plants, causing fruit blemish and premature drop that result in severe economic losses in commercial citrus orchards. However, CBS symptoms and effects may vary depending on the citrus species: *Citrus limon* (lemon) is susceptible and highly affected by the disease, while no CBS-related damage has ever been observed for *Citrus latifolia* (Tahiti lime), implying that it must be resistant to the disease. The difference in the response to this disease provided the opportunity to gain insight into the metabolites responsible for the resistance by comparison of the metabolomic profiles of these two citrus species. Metabolic variations of *C. limon* and *C. latifolia* inoculated with *P. citricarpa* were analyzed using various metabolomic-based platforms including ^1^H NMR for overall metabolic profiling, and LC-MS and HPTLC for targeted analysis. The ^1^H NMR spectra of the samples demonstrated that certain phenolics were strongly induced after pathogenic inoculation, especially in the resistant species. The induced phenolics were identified from *C. latifolia* by further ^1^H NMR, LCMS and HPTLC analysis yielding six prenylated and methoxy coumarins, i.e., 5,7-dimethoxycoumarin, 5-geranyloxy-7-methoxycoumarin, 7-geranyloxycoumarin, 8-methoxypsoralen, 5,8-dimethoxypsoralen and 5-geranyloxypsoralen. These isolated coumarins and a coumarin-rich fraction were tested against the fungal pathogen, *P. citricarpa*, to evaluate their activity. None of the individual coumarins exhibited a significant inhibition, while the coumarin fraction exhibited a strong antifungal activity suggesting a synergistic interaction of its components. To obtain further insight into the roles of these compounds in the plant defense, the possible mechanisms of the individual coumarins were tested using an *in-silico* model, the PASS Online Tool; the analysis showed that each coumarin appeared to have a unique defense mechanism, even with very slight variations in the chemical structures. The results could provide evidence of the existence of a complex plant defense mechanism consisting in a multitude of synergistic interactions between compounds.

## Introduction

The Citrus species is renowned for the attractive organoleptic features of its fruit, whose pulp and juice are among the most vastly popular throughout the world (Wu et al., 2018; Wang et al., 2019). New citrus varieties of citrus species are continually being developed and introduced into the market to follow the trends of customers’ preferences. The commercial value of citrus fruit is not limited to its pulp and juice, since their peel and pericarp are an interesting source of products with high market value such as molasses, pectin, volatile organic compounds and oils among others (Bocco et al., 1998; Anticona et al., 2020).

The world production of citrus has increased rapidly from 34.9 million tons in 1968 to 146 million tons in 2017, for example, due to their large market demand. China is currently the largest producer of citrus fruit worldwide, with an annual production of around 42.8 million tons, followed by Brazil (19.6 tons), India (12.04 tons) and Mexico (8.1 tons) (NationMaster, 2020). Notably, in Brazil, most of the production of oranges is used for juice processing and exported to several countries, such as the European Union (∼65.7%), United States (∼21.1%), Japan (∼5.1%), China (∼4.5%) and others (∼3.6%) (Citrusbr, 2020). The main citrus belt of Brazil is located in São Paulo and Minas Gerais states, which produced around 268.63 million 40.8 kg-boxes of oranges in the 2020/2021 season (Fundecitrus, 2021). These numbers reflect the importance of citrus production in terms of social and employment relevance in Brazil (Neves et al., 2010).

Similarly to most crops, citriculture often faces severe phytosanitary problems which can lead to losses (Neves et al., 2010). In the main Brazilian citrus belt, several diseases including citrus black spot (CBS) caused by the fungus *Phyllosticta citricarpa* (Silva Junior et al., 2016) and huanglongbing (HLB) by *Candidatus liberibacter* spp. (Bassanezi et al., 2011), or citrus canker caused by the bacteria *Xanthomonas citri* subsp*. citri* (Behlau, 2020), have been responsible for severe damage in citrus orchards. Among the diseases, CBS is one of the most serious in the tropical and subtropical citrus-growing regions of Africa, Asia, Oceania, and the Americas (EFSA 2014; Kotzé 2000; (Silva Junior et al., 2016), which likely provide optimum conditions for the proliferation of this disease (Yonow et al., 2013; EFSA 2014). While the damage produced by CBS does not destroy the citrus orchards, it can deteriorate the quality of fruit peel but the damage is restricted to the flavedo and does not necessarily affect juice quality (Kotzé, 1981). However, the fruit blemishes in susceptible species reduce their marketability and premature fruit drop results in a loss in yields of up to 80% (Silva Junior et al., 2016).

*Phyllosticta citricarpa* infects a wide range of citrus species and varieties, including oranges, tangerines, and lemons with significant qualitative and quantitative damages (Goes, 1998); Tran et al., 2018). Commercial citrus, with the exception of Tahiti lime (*C. latifolia*), are considered to be susceptible to CBS. Interestingly, in areas with high counts of *P. citricarpa* inoculum, the pathogen has been isolated from the asymptomatic leaves and fruit of Tahiti lime in Brazilian orchards (Baldassari et al., 2007). On the other hand, lemon (*C. limon*) is considered one of the most susceptible species to *P. citricarpa* and the CBS symptoms are frequently observed on their fruits and leaves (Kotzé, 1981; Truter et al., 2007).

The main CBS management strategy adopted during the critical period of infection (Lanza et al., 2018). However, given the increasing restrictions to the use of these chemicals, there is a growing need for the development of more acceptable alternatives (Gullino & Kuijpers, 1994; Ragsdale & Sisler, 1994). These alternative methods include exclusion practices (quarantine and sanitation), and other genetic, and biological methods as part of a more integrated approach to control CBS (Silva Junior et al., 2016; Dewdney et al., 2018; Guarnaccia et al., 2019), Rodriguez et al., 2018).

The investigation of plant defense mechanisms that are effective against certain pests or enemies could potentially reveal metabolites or more likely, whole systems that can then be used on plants that lack these resources (Pino et al., 2013). Given its current importance, a great deal of research has been published on this topic and specialised metabolites such as D-limonene, ursolic acid, 19α-hydroxy-8,11,13-abietatriene (dehydroabietinol), hesperidin, tangeretin and sinsentin have been reported to exhibit pesticide or fungicide activities (Rodriguez et al., 2018, Ortuño & Del Río, 2009; Santos et al., 2010; Cespedes et al., 2014). In the case of citrus species, several phytoalexins and phytoanticipins have been associated with plant pathogen control. In particular, coumarins such as scoparone (5,6-dimethoxycoumarin), scopoletin (6-methoxy-7-hydroxycoumarin), xanthyletin (6,7-dimethylpyranocoumarin), umbelliferone (7-hydroxycoumarin), bergapten (5-methoxypsoralen), and citropten (5,7-dimethoxycoumarin) have shown *in vitro* antifungal activity against different fungus pathogens, such as *Penicillium digitatum*, *Phytophthora citrophthora* and *Colletotrichum* sp. (Khan et al., 1985; Kim et al., 1991; Afek et al., 1999; Sanzani et al., 2014; Ramirez-Pelayo et al., 2019).

In the era of systems biology, chemical profiling is the key step in life sciences research for metabolism-related topics. Successful and representative metabolic profiling relies on the range of detected metabolites and the sensitivity of the selected analytical platforms for their detection (Wolfender et al., 2010). Due to the inherent limitations of any of the currently available analytical methods, the integration of various analytical techniques is essential to obtain comprehensive information on the large range of metabolites present in a crude extract (Gaudencio & Pereira, 2015). Many metabolomic studies have been performed using multiplatform technologies, including ^1^H NMR, LCMS, HPLC and GCMS (Allwood et al., 2011). Among these and despite its relatively low sensitivity and resolution, ^1^H NMR is very often applied to obtain a general profile of metabolites that may eventually provide information for a chemical fingerprint that requires robustness/reproducibility of data and a broad metabolome overview. Furthermore, the low sensitivity of NMR methods can be compensated by additionally applying MS-based hyphenated technologies, GC-MS being particularly useful for primary metabolites and LCMS for specialized (secondary) metabolites (Wolfender et al., 2013). High performance thin-layer chromatography (HPTLC) has also been used as a supplementary tool for metabolomics, allowing the highly robust visualization of specific groups of metabolites. Its efficient application for the chemical fingerprinting of plants has resulted in its adoption for the quality control of many medicinal plants (Maldini et al., 2016; Salome-Abarca et al., 2018; Maldini et al., 2019; Salome-Abarca et al., 2021).

An additional advantage of HPTLC as a metabolomics tool lies in its facilitation of the isolation of compounds. This step is essential in a metabolomics study since even when the identification of significant metabolites is done initially by comparison with databases, the confirmation or in some cases, the identification itself requires the full spectral analysis of pure samples. In this case, HPLTC has proved to be an efficient preparative technique (Salome-Abarca et al., 2018; Salome-Abarca et al., 2021) due to distinctive features such as easy chemical fingerprinting aided by the detection of a broad range metabolites thanks to the use diverse chemical visualization reagents if needed. In the case of phenolics, including coumarins characterized by their high UV absorption and fluorescence, HPTLC fingerprinting can provide interesting information with the additional advantage of allowing quick and simple *in situ* isolation of metabolites of interest (Salome-Abarca et al., 2018).

The aim of this study was to identify differences in the metabolomic make-up of two citrus species, *C. limon* and *C. latifolia*, which could be responsible for their respective susceptibility and resistance to black spot disease. For this, leaf extracts from both citrus species inoculated with *P. citricarpa* were submitted to a metabolomics study using a combined ^1^H NMR, LC-MS and HPTLC analytical platform. This allowed the selection of putative metabolites associated with plant resistance which were identified by targeted analysis. In addition, the possible mechanisms of fungicide action of the isolated compounds were deduced from *in-silico* studies.

## Materials and Methods

### Pant Material and Inoculation

Six-months-old *C. limon* and *C. latifolia* samples were obtained from a commercial citrus nursery. Inoculation of leaves was done by spraying them with *P. citricarpa* at a concentration of 10^5^ conidia/ml. Non-inoculated leaves were used as controls. The inoculated and non-inoculated leaves were collected at the inoculation time and then 1, 15, and 60 days after inoculation, immersed in liquid nitrogen and immediately freeze-dried. Five replicates were obtained for each data point (five plants).

### ^1^H NMR

Samples were prepared by ultrasonication of 30 mg of freeze-dried leaves with 1 ml of CH_3_OH-*d*_*4*_ containing 3.93 mM hexamethyldisiloxane (HMDSO) as an internal standard, for 15 min at 25°C. After centrifugation at 13,000 rpm, 300 µl of the supernatant were transferred into 3 mm NMR tubes for ^1^H NMR analysis. The ^1^H NMR experiments were performed on an AV-600 MHz NMR spectrometer (Bruker, Karlsruhe, Germany), operating at a frequency of 600.13 MHz at 25°C; CH_3_OH-*d*_*4*_ was used as a locking solvent. The acquisition parameters were set as follows: pulse program (PULPROG) zgpr30, acquisition time (AQ) 2.72 s, recycle delay (D1) 1.5 s, number of scans (NS) 64, pulse width (P1) 7.5 μsec (90°), mixing time 0.010 s, receiver gain (RG) 287, spectral width (SW) 20 ppm, size of fid TD (F1) 65.53, size of real spectrum (SI) 65.53, exponential line broadening 0.3 Hz. A pre-saturation sequence was used to suppress the residual water signal, using low power selective irradiation at H_2_O frequency during the recycle delay. The resulting spectra were manually phased, and the baseline was corrected and calibrated to the HMDSO signal at 0.06 ppm using TOPSPIN V. 3.0 (Bruker) program.

The NMR spectra were bucketed using AMIX 3.9.12 (Bruker Biospin GmbH Rheinstetten, Germany). The bucketed data was obtained by integration of the spectra at 0.04 ppm intervals. The peak intensity of individual peaks was scaled to the total intensity recorded from δ 0.30 to δ 11.50. Due to the residual signals of H_2_O and CH_3_OH-*d*_*4*_, the regions δ 4.7–δ 4.9 and δ 3.28–δ 3.34 were excluded from the analysis.

Databases, such as HMDB (Human Metabolome Database), BMRB (Biological Magnetic Resonance Data Bank), SDBS (Spectral Data Base for Organic Compounds) and a home-made database were used for metabolite characterization.

Multivariate analysis was performed using SIMCA P (version 15.1, Umetrics, Umeå, Sweden). Principal component analysis (PCA) was used to examine intrinsic variations in the dataset. All the raw data was Pareto-scaled. The variables were subjected to an orthogonal projection to latent structures discriminant analysis (OPLS-DA) to identify differential components among samples. The quality of the model was estimated by R^2^X and Q^2^ values. Q^2^ values were obtained by permutation tests (100). R^2^X indicated the fitness of model and was defined as the proportional variance, whereas Q^2^ was defined as the predictable variance (Villa-Ruano et al., 2019). Moreover, the analysis of variance testing of cross-validated predictive residuals (CV-ANOVA) was used to access the reliability of the supervised models, *p*-values < 0.05 are considered as significant and *p* < 0.01 as highly significant.

### LCMS

For metabolomics analysis by LCMS, 30 mg freeze-dried plant material were ultrasonicated for 20 min with 1 ml of CH_3_OH, and then centrifuged at 13000 rpm; 100 µl of the supernatant were transferred to vials containing 900 µl of CH_3_OH. Quality control (QC) samples were prepared by mixing 50 µl of each sample in a vial. The analysis was performed using an UHPLC-DAD chromatograph (Thermo Ultimate 3000) connected to a O-TOF-Q II spectrometer with electrospray ionization (ESI) (Bruker). The separation was performed using a Kinetex, C18 column (2.1 × 150 mm, 2.6 μm, Phenomenex) and eluted using a gradient of water (A) and acetonitrile with 0.1% formic acid (B) (5–98% B in 32 min). The column temperature was maintained at 35°C. The flow rate was 0.3 ml/min and the injection volume was 1 µl. The mass spectrometer parameters were set as follows: nebulizer gas 2.0 bar, drying gas 10.0 ml min^−1^, gas temperature 250°C, capillary voltage 3500 V; spectra were obtained in the positive mode, in a 100–1650 m/z range. Samples were injected in a random sequence, and QC samples were injected every 10 samples. Characterization of phenolic compounds in the coumarins fraction of *C. latifolia* was performed with target analysis by using LC-QTOF (Agilent Technology 6545) in auto MS/MS mode with MassHunter Workstation software (version B.08, Agilent Technology). The separation was performed using a SB ODS C-18 analytical column (3.0 × 50 mm id. 1.8 μm particle) and eluted with a gradient of water (A) and acetonitrile (B) with 0.1% formic acid (5–100% B in 30 min). The flow was of 0.35 ml/min and the injection volume was 4 µl. The column temperature was maintained at 35°C. The spectra were obtained in the positive ionization mode in the following conditions: nebulizer gas at 60 psi, drying gas flow rate at 13 ml/min and temperature 300°C. The capillary voltage was set at 3000 V. Data was acquired in centroid mode. The full scan was carried out on three spectra in a 100–1700 *m/z* range.

For metabolomics analysis, the total ion chromatograms of all the samples were extracted and the acquired raw MS files were processed with DataAnalysis (Bruker). XCMS software was used for data pre-treatments including peak identification, peak alignment, peak feature extraction, and peak area normalization. The mass spectrometry matrix data containing sample names, *m/z*-retention time pairs, and ion intensity information were generated and exported. Any background ions were removed by comparison with blank samples, and the quantitative results were normalized with QC samples. Finally, the qualitative and quantitative data were used for subsequent multivariate analysis (MVDA) performed using SIMCA P (version 15.1, Umetrics, Umeå, Sweden).

The targeted analysis of metabolites in the coumarin fraction allowed the tentative identification, based on their MS/MS spectra and the comparison with databases (METLIN MS and MSMS, MassBank MSMS) and literature.

### High Performance Thin-Layer Chromatography

For HPTLC analysis, 30 mg freeze-dried plant material were ultrasonicated for 20 min with 1 ml of CH_3_OH and then centrifuged at 13000 rpm; 100 µl of the supernatant were transferred into vials containing 900 µl of CH_3_OH. HPLTC analyses were performed on a CAMAG HPTLC system equipped with an automatic TLC sampler (version ATS4), derivatiser (version 1.0 AT), TLC plate heater (version III) and TLC visualizer (CAMAG, Muttenz, Switzerland). Ten microliters corresponding to day-1 and day-60 samples of non-inoculated and inoculated leaves from *C. limon* and *C. latifolia* were spotted in 6 mm bands on 20 × 10 cm silica gel 60F_254_ plates (MerckMillipore, Darmstadt, Germany). Samples were spotted at 10 mm from the bottom edge and 20 mm from the left and right borders of the plate (2 species/2 inoculation/2 × day/2 replicates, totaling 16 samples). The distance between the bands was 8.8 mm. Two mobile phases were used for HPTLC analyses; a polar phase containing EtOAc-formic acid-acetic acid -H_2_O (100:11:11:27, v/v/v/v) and a non-polar phase consisting of toluene-EtOAc (8:2, v/v). The chamber saturation time was 20 min and solvent migration distance was 80 mm from the application point. Developed HPTLC plates were sprayed with 2 ml of 2-aminoethyl diphenylborinate solution using automatic derivatization. Images of the derivatized plates were recorded using a TLC visualizer at 366 nm before and after derivatization.

### Extraction and Isolation of Target Compounds From *C. latifolia*

Target compounds obtained from *C. latifolia* were isolated using *Pure C-850 FlashPrep chromatography* (Buchi, Flawil, Switzerland). Samples of 26 g of dried and powdered leaves were sonicated for 30 min with 95% aqueous methanol at room temperature and filtered. Resulting extracts were dried, yielding a 2.10 g residue. Separation and isolation of compounds was achieved by injection of samples into a 40 × 63 µm column with 80 g silica gel and eluted at 25 ml/min using a gradient of CH_3_OH (A) and CHCl_3_ (B) (5–100% B in 55 min), followed by 5 min equilibration time in initial conditions. Seven fractions were collected and analyzed by TLC. The fraction containing coumarins (200 mg) was further purified on a 40 × 63 µm column with 12 g silica gel and eluted at 10 ml/min with a gradient of *n*-hexane (A) and EtOAC (B) (0–100% B in 65 min) and 4 min equilibration time to initial conditions. This yielded 1.0 mg of compound **1**. The same fraction was reinjected into the same column eluted at 10 ml/min but using the gradient of toluene (A) and EtOAC (B) (0–100% B in 53 min) and 5 min equilibration time to initial conditions. This yielded 0.85 mg of compound **2**, 0.58 mg of compound **3**, and 1.75 mg of compound **4**.

The isolated compounds were dissolved in 300 µl of CH_3_OH-*d*_*4*_ and transferred to 3 mm NMR tubes for ^1^H NMR analysis.

The compound 1 (5,7-dimethoxycoumarin) was quantified using the ratio between its methoxy protons signal (δ 3.93, s, 3H) and the internal standard (HMDSO) signal (δ 0.06).

### Antifungal Activity Against *Phyllosticta citricarpa*

The assays were performed with an isolate of *Phyllosticta citricarpa* obtained according to Clinical and Laboratory Standards Institute (CLSI, 2002). The pathogen was cultivated in potato dextrose agar (PDA) for 14 days at 25°C. After this period, 1 ml of saline solution (NaCl 0.85% w/v) was added to the colony and homogenized. The spore suspension was transferred to a tube and shaken for 5 min, after which the supernatant was measured by UV-VIS spectrophotometer at 625 nm. The suspension was diluted in saline solution to obtain an absorbance value of 0.1 and then further diluted 1:50 in potato dextrose broth (PDB) to obtain a concentration equivalent to twice the density required for antimicrobial tests (5 × 10^4^ CFU/ml).

### Microbial Growth Kinetics

The growth kinetics of the microorganism was determined before performing the antimicrobial assays. A mixture of 100 µl of *P. citricarpa* suspension and 100 µl of PDB were placed in each well of a 96-well plate to observe the pathogen growth; a sample of 200 µl of the broth was used as a negative control. The plate was incubated for 7 days at 25°C and 625 nm absorbance was monitored every six hours.

### Determination of Fungicidal Activity

The fungicidal activity of the coumarin fraction (CF) and the isolated and identified compounds from *C. latifolia* (**1**: 5,7-dimethoxycoumarin; **2**: 8-methoxypsoralen; **3**: 5,8-dimethoxypsoralen; **4**: 8-geranyloxypsoralen; **6**: 5-geranyloxy-7-methoxycoumarin) was determined using the microdilution method (CLSI, 2002). Compound 6,5-geranyloxy-7-methoxycoumarin was isolated from peels of *C. latifolia* while the other compounds were purchased from Sigma-Aldrich. The coumarin fraction and the isolated compounds were dissolved in DMSO (2% in PDB, v/v), to concentrations of 4 mg/ml and 1 mg/ml, respectively. These solutions were then diluted 1:2 in PDB. For the assay, 100 µl of the fungi suspension (5 × 10^4^ CFU/ml) were placed in each well plate and incubated for 120 h at 25°C. After that, 100 µl of the different compound solutions were added to each well plate. Well plates containing only PDB or PDB with the pathogen were used as controls. Aliquots of 100 µl DMSO (100%) or DMSO (2%) were also used as controls.

### *In-Silico* Analysis

*In-silico* analysis of the compounds isolated and identified from *C. latifolia* by LC-MS/MS were performed using the program Prediction of Activity Spectra for Substances- *PASS online* (Filimonov et al., 2014) to predict their fungicidal mechanism of action. This analysis compares the chemical structure of substances of interest with those of biologically active compounds available in a database. The results were expressed as the difference in the probabilities of each compound to be active (Pa) and inactive (Pi) for each mechanism of action investigated and were classified as low, moderate, and high potential according to Seibert et al. (2019).

## Results

### ^1^H NMR Analysis of Citrus Leaves

**FIGURE 1:**
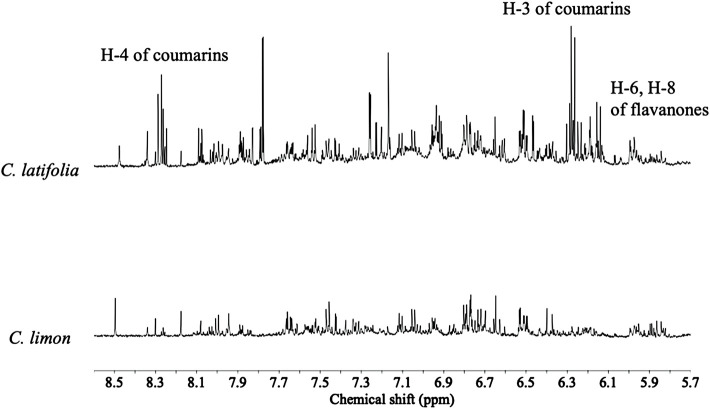
Typical ^1^H NMR (600 MHz in CH_3_OH-*d*_*4*_) spectra of *Citrus latifolia* and *Citrus limon* species in the range of δ 5.0–8.5.

**FIGURE 2:**
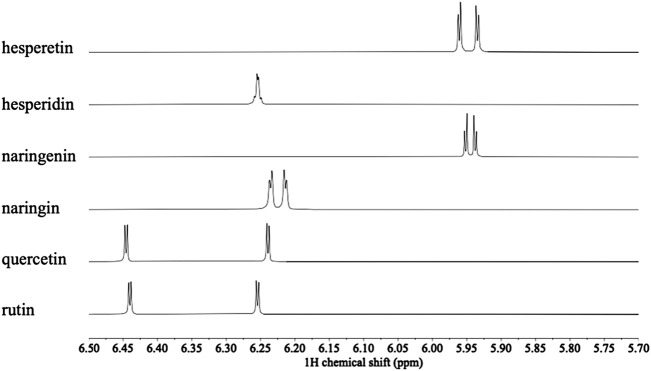
Comparison of ^1^H NMR spectra of hesperetin, hesperidin, naringenin, naringin, quercetin and rutin (600 MHz in CH_3_OH-*d*_*4*_) in the range δ 5.7–6.5 for H-6 and H-8.

Overall ^1^H NMR chemical profiles of two citrus species were compared. The metabolic profiles of the species were distinguishable, especially in the characteristic range for phenolics, i.e., δ 6.0–δ 8.6 (Figure 1). In this region two set of doublets (8.8 Hz) characteristic of flavonoids with a disubstituted B-ring were detected at δ 7.77, δ 7.79, and δ 7.82 (H-3′ and H-5′), and δ 7.04 and δ 7.11 (H-2′ and H-6’). The resistant species showed a higher intensity of coumarins and flavanones in the ^1^H NMR spectra. Coumarin signals were also clearly detected as characteristic doublets (9.6 Hz) at δ 6.1–6.3 (H-3), and δ 8.1–8.3 (H-4). In addition, two doublets (1.8 Hz) corresponding to H-6 and H-8, characteristic of flavanones (e.g., hesperetin and naringenin), which are shifted upfield from those of flavones at δ 6.2–6.5 to around δ 5.9 (Figure 1) were also detected. Compound identities were confirmed by comparison of the ^1^H NMR spectra of reference compounds (Figure 2). All the identified primary metabolites are listed in Supplementary Table S1 together with the information of their ^1^H chemical shifts.

For further insight on metabolites that could plausibly be associated with citrus resistance to CBS, the ^1^H NMR data was submitted to a multivariate data analysis, classifying the plant samples in two classes: control and infected *C. limon* and *C. latifolia* leaves, and four time points for each (0, 1, 15 and 60 days after inoculation).

**FIGURE 3:**
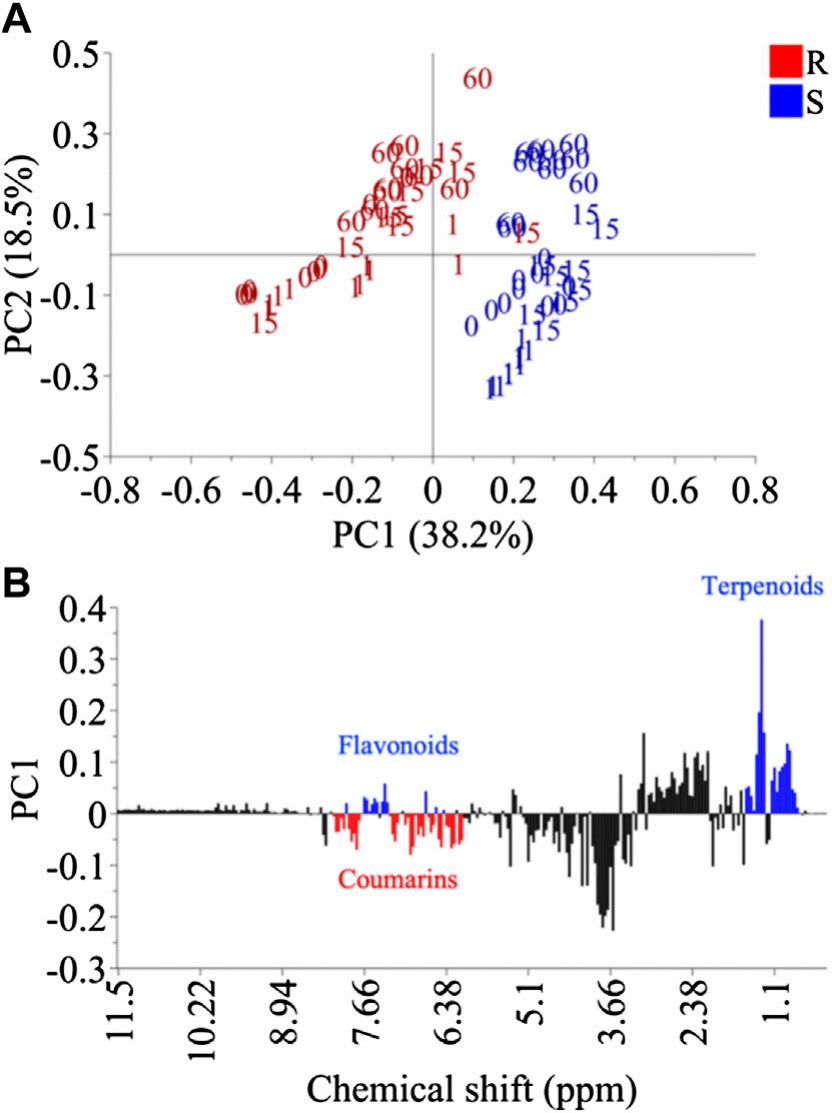
Principal component analysis of *Citrus limon* (S) and *Citrus latifolia* (R). Score plot using PC1 and PC2 **(A)** and loading plot of PC1 **(B)**.

Principal component analysis (PCA) was firstly carried out for the dataset. The resulting model showed 96.4% variation with 13 PCs. When the data was analyzed together, the PCA plot did not show a clear separation between inoculated and non-inoculated samples. The main separation along the PC1 was due mainly to species (PC1) and developmental stage (PC2) (Figure 3A). The metabolic difference between the resistant (*C. latifolia*) and susceptible (*C. limon*) species lay in three specific metabolic groups regardless of whether they were or not inoculated: flavonoids, coumarins and terpenoids (Figure 3B). The leaves of *C. latifolia* were found to have relatively higher amounts of coumarins than those of *C. limon*, indicating that they could be related to the resistance observed for *C. latifolia*. However, the connection of coumarins to the resistance could not be confirmed because the metabolic differentiation of the fungus-infected plants was not distinguished in the PCA. The influence of intrinsic species and age effects were apparently major variations that could override the fungal inoculation effect. Therefore, using class data (control and inoculation) to discriminate species effect and inoculation time as Y-data, a supervised discriminant analysis, orthogonal partial least square analysis (OPLS) analysis was applied to the ^1^H NMR data set of each species, separately.

**FIGURE 4:**
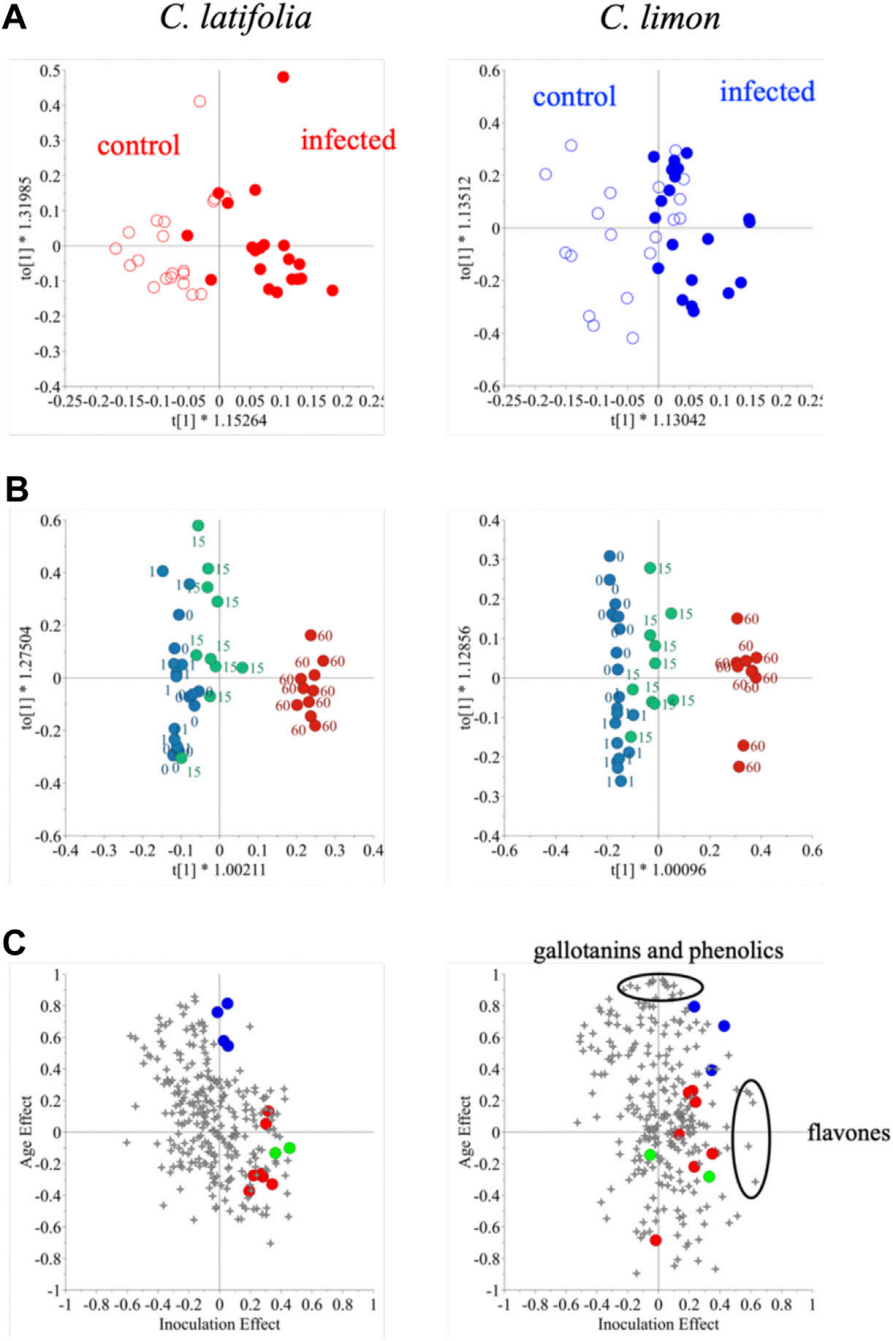
Orthogonal partial least square analysis – discriminant analysis of ^1^H NMR spectra of *Citrus latifolia* and *Citrus limon* for the inoculation effect **(A)** orthogonal partial least square analysis of the same dataset for the days post inoculation (dpi) effect **(B)**, and shared and unique structures (SUS)-plot for the inoculation (X-axis) and dpi (Y-axis) effect **(C)** of the same dataset. In Figure 4C, malic acid (•). flavanones (•), coumarins (•).

OPLS was applied to the effect of inoculation and days post inoculation (dpi) individually. To test for the inoculation effect, qualitative data (inoculation and control) was used for OPLS-discriminant analysis (DA) while continuous quantitative data (days) was used to analyze the dpi effect. The resistant species, *C. latifolia* was well separated by inoculation but this effect was much lower in the susceptible species *C. limon* (Figure 4). The Q^2^ values of resistant and susceptible species were 0.42 and 0.21, respectively and *p*-values < 0.01 and >0.05, respectively. This meant that the OPLS-DA model of *C. limon* species was not validated though there some discrimination appeared after 15 days of inoculation (Figure 4A). As for dpi both species were well distinguished an effect that was clearly evident especially 60 days after inoculation (Figure 4B).

Thus, changes in citrus metabolome seemed to be affected by two factors: inoculation and age. To investigate the effect on the metabolome in detail, the OPLS-DA models were combined, and the generated shared and unique structures (SUS)-plot was further interpreted (Figure 4C). The *X* and *Y*-axes correspond to the effect of inoculation and dpi so that the ^1^H NMR signals at top-center and far-right are associated solely with dpi and inoculation, respectively. The resistant species was found to accumulate more coumarins and flavanones, while malic acid was identified as the marker compound for age. In the case of susceptible *C. limon* leaves, as mentioned, the inoculation effect was not validated. Despite failing the validation given its low Q^2^ value and *p* value >0.05 when two OPLS and OPLS-DA models were combined in a SUS-plot, the metabolites related to the fungus infection were found to be completely different from those of *C. latifolia*; these metabolites induced by the inoculation were identified to be flavones while gallotannins and some phenolics were associated with age. Coumarins and flavanones in the inoculated susceptible species, however, were not strongly related to the fungus inoculation (Figure 4C). This might be due to their low concentration in *C. limon* or to an incomplete induction.

Due to the high degree of signal congestion, it was impossible to get detailed information on phenolic compounds. Thus, ultra-high-performance liquid chromatography (UHPLC) coupled to quadruple-time of-flight-mass spectrometry (UHPLC-QTOF-MS) was applied to the same sample set targeting the phenolics.

### UHPLC-QTOF-MS

The PCA analysis of UHPLC-MS data did not show any clear separation between inoculated and non-inoculated samples. Similarly to ^1^H NMR data, the separation was mainly species and developmental stage (dpi)- related. The UHPLC-MS data of the resistant and susceptible species was further analyzed by OPLS-DA to study the metabolic difference (Supplementary Figure S1A), showing them to be highly distinguishable Naturally, while their distinctive metabolic profiles could give indications regarding potential defense-related metabolites, it is unclear which of these differential metabolites are simply species-related rather than defense-related. The *S*-plot revealed [M + H]^+^ ions at *m/z* 207.0592, 339.1582, 217.0436, 247.0545 that were notably higher in the resistant species (Supplementary Figure S1B). These ions were predicted to be coumarin analogues by searching their molecular formula in a database.

For the identification of the metabolites, which was performed by LC-QTOF analysis, the detected ions were further analyzed by MS/MS analysis and compared with those in the database of MassHunter as well as Massbank and Dictionary of Natural Products 30.2. The MS/MS spectra are shown in (Supplementary Figures S5A–F).

From the MS and MS/MS data providing precursor and fragmented ions (Table 1) as well as the information from the database, six coumarins were dereplicated to be 5,7-dimethoxycoumarin (**1**), 8-methoxypsoralen (**2**), 5,8-dimethoxypsoralen (**3**), 5-geranyloxypsoralen (**4**), psoralen (**5**), and 5-geranyloxy-7-methoxycoumarin (**6**). Most fragments correspond to the loss of CH_3_ and geranyl groups.

**TABLE 1:** The identification of the metabolites higher in *Citrus latifolia* than *Citrus limon* by LC-QTOF-MS/MS analysis in the coumarin fraction.

No	Compound name	Molecular formula	RT	Molecular Weight	Precursor ion (*m/z*)	Main *m/z* fragments		
1	5,7-dimethoxycoumarin	C_11_H_10_O_4_	11.65	206.0579	207.0644	192.0414	149.0463	121.0648
2	8-methoxypsoralen	C_12_H_8_O_4_	12.02	216.0422	217.0493	202.0258	174.0308	161.0591
3	5,8-dimethoxypsoralen	C_13_H_10_O_5_	12.07	246.0582	247.0605	232.0367	217.0132	189.0185
4	5-geranyloxypsoralen	C_21_H_22_O_4_	20.91	338.1518	339.1590	203.0337	175.0442	147.0436
5	psoralen	C_11_H_6_O_4_	21.04	202.0266	203.0334	175.0389	147.0439	103.0537
6	5-geranyloxy-7-methoxycoumarin	C_20_H_24_O_4_	21.23	328.1674	329.1741	193.0509	137.1327	-

High-performance thin-layer chromatography (HPTLC) fingerprinting of Citrus latifolia and Citrus limon and isolation of target coumarins.

However, as mentioned before, it is essential to distinguish the defense-related metabolites in *C. latifolia* from other profiling differences. For this, the metabolites that were presumably induced by the fungal inoculation should also be analyzed as phytoanticipins type metabolites. Thus, an, OPLS-DA model was built with data from the resistant species, *C. latifolia,* to gain an insight into the metabolites associated with the resistance after the fungal inoculation. Interestingly, contrary to the results of the OPLS-DA analysis of ^1^H NMR shown in Supplementary Figure S1, the metabolic difference in the inoculated leaves of *C. latifolia* was not detected till 60 days after inoculation, that is, the OPLS-DA model in early stages with Q^2^ values were below 0.2, and thus could not be validated the first stages. The difference was shown only in the samples 60 days after the infection (Supplementary Figure S2A). The S-plot of the UHPLC-DAD-MS analysis revealed a couple of mass features in the inoculated samples of *C. latifolia* (Supplementary Figure S2B). The ions at *m/z* 203.0292 and at *m/z* 339.1581 were characterized as psoralen (**5**) and 5-geranyloxypsoralen (**4**), respectively. Along with these compounds, an ion with *m/z* 282.2746 was clearly detected in the resistant species that was predicted to be a long chain aliphatic amino alcohol based on its molecular formula based on the molecular formula (C_18_H_36_NO). However, the same ion was detected in the susceptible species, *C. limon*, indicating that the metabolite is not resistance-related but rather a chemical marker of fungal infection or a fungal metabolite of *P. citricarpa*.

While UHPLC-QTOF-MS and LC-QTOF-MSMS analysis provided chemical details of the samples. High-performance thin layer chromatography (HPTLC) fingerprinting analysis was performed on the extracts of both citrus species in use of coumarin’s characteristic intense blue florescence.

The HPTLC separation with the mobile phase of toluene-EtOAc (8:2, v/v) showed a clear differentiation between *C. limon* and *C. latifolia* specially in the R_f_: 0.4–0.55 range (Supplementary Figure S3). In this region, the bands with blue florescence at 365 nm showed far higher intensity in *C. latifolia* than in *C. limon*. The characteristic color and high fluorescent emission corroborated the presence of coumarins, in accordance with NMR and LC-MS data. Based on the HPTLC chromatograms, the compounds were isolated by medium pressure chromatography (MPLC).

The chromatographic separation allowed the isolation of four compounds, three of which were fully elucidated by 1D and 2D NMR while the fourth was partially elucidated. Compound **1** was identified as 5,7-dimethoxycoumarin (citropten). Two characteristic resonances of pyrone ring were detected at 6.15 (H-3, d, *J* = 9.6 Hz, 1H) and δ 8.09 (H-4, d, *J* = 9.6 Hz, 1H) which were correlated by ^1^H-^1^H-correlated spectroscopy (COSY). The additional signals at δ 6.47 (H-6, d, *J* = 2.2 Hz, 1H) and δ 6.52 (H-8, d, *J* = 2.2 Hz, 1H) were assigned to the protons in the aromatic ring, and signals at δ 3.87 (s, 3H) and δ 3.93 (s, 3H) were assigned to methoxy protons and confirmed by HMBC correlations observed at δ 157.4 (C5) and 164.5 (C7), respectively. Compound **2** (5-geranyloxy-7-methoxycoumarin) and **3 (**7-geranyloxycoumarin) showed the distinctive protons of a pyrone ring and geraniol moiety. The resonances at δ 6.15 (H-3, d, *J* = 9.5 Hz, 1H) and δ 8.08 (H-4, d, J = 9.5 Hz, 1H) for compound **2** and δ 6.28 (H-3, d, *J* = 9.6 Hz, 1H) and δ 8.26 (H-4, d, *J* = 9.6 Hz, 1H) for compound **3** were detected in ^1^H NMR spectra. The geraniol moiety of compound **2** was observed at δ 4.70 (H-1a′, 1b′, d, *J* = 6.74 Hz, 2H), δ 5.49 (H-2′, t, *J* = 6.74 Hz, 1H), δ 5.09 (H-6′, t, *J* = 6.03 Hz, 1H), δ 2.10 (H-4′, m, 2H) and δ 2.15 (H-5′, m, 2H) as well as three methyl resonances at δ 1.60 (H-10′, s, 3H); δ 1.64 (H-8′, s, 3H) and δ 1.78 (H-9′, s, 3H). In the case of compound **3**, the resonances of the geraniol moiety were assigned at δ 5.03 (H-1a′, 1b′, d, *J* = 7.30 Hz, 2H), δ 5.54 (H-6′, t, *J* = 7.30 Hz, 1H), δ 2.05 (H-4′, m, 2H) and δ 2.07 (H-5′, m, 2H) as well as δ 1.67 (H-9′, s, 3H), δ 1.62 (H-10′, s, 3H), and δ 1.57 (H-8′, s, 3H). The ^1^H NMR assignments together with COSY and HMBC correlations and comparison with literature (Miyake and Hiramitsu, 2011; Nakatani et al., 1987) confirmed compounds **2** and **3** to be 5-geranyloxy-7-methoxycoumarin and 7-geranyloxycoumarin (auraptene), respectively. Compound **4** unfortunately was not fully elucidated due to signal overlapping. Also, the signals did not show clear correlations in the 2D NMR spectra. Nonetheless, characteristic signals and correlations suggested a furanocoumarin-like structure. In these spectra, the characteristic signals of an AB system in a lactone ring of coumarin were also observed at δ 6.27 (H-3, d, J = 9.6 Hz, 1H), δ 8.27 (H-4, d, J = 9.6 Hz, 1H), 6.28 (d, J = 10.1 Hz, 1H), and 8.26 (d, J = 10.1 Hz, 1H). Signals from a geranyloxy group also appeared at δ 5.03 (H- 1a′, 1b′, d, 6.84 Hz, 2H), δ 5.54 (H-2′, t, 6.84 Hz, 1H), δ 4.14 (H-6′, t, 6.61 Hz, 1H), δ 2.01 (H-4′, m, 2H) δ 2.09 (H-5’, m, 2H). Methyl groups signals were clearly observed as singlets at δ 1.64, δ 1.66 and δ 1.68. Altogether this suggests a prenylated coumarin-like compound. Another option was that compound **4** was a mixture of two coumarins. All the NMR spectra are shown in (Supplementary Figures S6A–M).

### Antifungal Activity of Isolated Coumarins From *Citrus latifolia* Against *Phyllosticta citricarpa*.

Since *P. citricarpa* possesses a slow growth in the initial growth stage, a growth kinetic model was built to determine the initiation of its log growth phase, in which the cell mass and number of cells increase exponentially. Growth variation was visible 120 h after incubation and the differences in optical density (OD) could be followed in the next few hours. Using this preliminary test, the inoculum incubation time was fixed at five days with subsequent compound treatment for 24 h.

Unfortunately, none of the tested compounds (Supplementary Figure S4) showed strong antifungal activity against *P. citricarpa.* If any, 5,7-dimethoxycoumarin (**1**) and 8-methoxypsoralen (**2**) showed a mild inhibition against the pathogen with a MIC = 500 μg/ml, while the other compounds, 5,8-dimethoxypsoralen (**3**); 8-geranyloxypsoralen (**4**); 5-geranyloxy-7-methoxycoumarin (**5**) showed a MIC >500 μg/ml. However, the coumarin fraction (which contains all the compounds) exhibited a MIC = 250 μg/ml.

To further study the potential effects and antifungal mechanisms of the *C. latifolia* coumarins, the compounds (Supplementary Figure S4) identified in the coumarin extract and tested against *P. citricarpa* were subjected to the Prediction of Activity Spectra for Substances (PASS) online tool for *in silico* activity analysis. All the potential mechanisms of action for the five compounds are listed in Table 2. These analyses are based on quantitative structure-activity relationship (QSAR) models (Seibert et al., 2019). All the tested compounds showed a antifungal potential but through different mechanisms suggesting a possible synergistic interaction among them.

**TABLE 2:** *In-Silico* test prediction of potential mechanism of action using the PASS online tool (Filimonov et al., 2014).

Potential mechanism of action	Pa-pi value				
	1	2	3	4	5
Cell wall biosynthesis inhibitor	0.283	0.246	0.246	-	-
DNA synthesis inhibitor	0.027	0.037	0.061	-	-
Protein synthesis inhibitor	0.145	0.108	0.133	0.159	0.165
Membrane permeability enhancer	0.467	0.138	0.237	0.458	0.530
Protein 30S ribosomal subunit inhibitor	-	-	-	-	-
Protein 50S ribosomal subunit inhibitor	0.006	0.016	0.016	-	-
DNA directed RNA polymerase inhibitor	-	0.023	-	0.107	0.087
NAD(P)^+^-arginine ADP-ribosyltransferase inhibitor	0.426	0.198	0.281	0.095	0.120
2-dehydropantoate 2-reductase inhibitor	0.549	0.314	0.223	-	0.032
Mycothiol-*S*-conjugate amidase inhibitor	-	-	-	-	-
Peptidoglycan glycosyltransferase inhibitor	0.300	0.119	0.119	0.003	0.028
CDP-glycerol glycerophosphotransferase inhibitor	0.650	0.491	0.651	0.709	0.695
Lanosterol 14 alpha demethylase inhibitor	-	-	-	-	-
CYP51 inhibitor	-	-	-	-	-
Squalene epoxidase inhibitor	0.127	0.041	0.015	0.135	0.196

**1:** 5,7-dimethoxycoumarin; **2**: 8-methoxypsoralen; **3**: 5,8-dimethoxypsoralen; **4**: 8-geranyloxypsoralen; **5**: 5-geranyloxy-7-methoxycoumarin. (-) Not indicated or unsatisfactory. (Pa - Pi) < 0.2: low potential; 0.2 ≤ (Pa - Pi) < 0.5: moderate potential; (Pa - Pi) ≥ 0.5: high potential.

## Discussion

Citrus plants appear to accumulate a series of phenolic compounds that might be involved in natural defense mechanisms. These metabolites could play an important role in defense activities or eventually may be precursors for defense systems. It is well known that in plant–microbe interactions, phenolic compounds also play a role in signaling. In line with this idea, several studies have reported that flavonoids and coumarins may act as phytoalexins and phytoanticipins in the defense mechanisms of citrus species against pathogen infection (Ortuño & Del Río, 2009; Ortuño et al., 2011; Ballester et al., 2013). The results of this study indicated that among phenolics, coumarins, play these roles in the defense mechanism of *C. latifolia* against *P. citricarpa*.

The basic structure of coumarins consists of two aromatic ring and in some cases an additional furan or pyran ring (Smyth et al., 2011). In the MS/MS spectra acquired in positive mode, the *m/z* 103.0537 and 91.0543 fragments correspond to the loss of CO_2_ from the pyrone ring system and to the protonated benzofuran of coumarins, respectively, and are commonly detected (Ledesma-Escobar et al., 2015). For ^1^H NMR analysis, the attachment of hydroxyl or alkoxyl groups causes a downfield shift of H-4 protons from δ 7.6–7.8 to δ 8.1–8.3, as in the case of bergapten (Kawaii et al., 1999), isoimperatorin (Fujioka et al., 1999), and phellopterin (Bergendorff et al., 1997).

A number of studies have described the antifungal effect of coumarins in citrus plants (Fracarolli et al., 2016; Ramirez-Pelayo et al., 2019). Among these, scoporane (5,6-dimethoxycoumarin) has been reported as the main phytoalexin of citrus produced as a defense against various plant pathogenic fungi, including *Phytophthora citrophthora* (Afek & Sztejnberg, 1988), *Guignardia citricarpa* (De Lange et al., 1976), *Botrytis cinerea* (Kuniga et al., 2015), *Penicillium digitatum* Sacc., *P. italicum* (Kim et al., 1991; Arras et al., 2006) and *Diaporthe citri* (Arimoto et al., 1986). Other coumarins such as scopoletin (6-methoxy-7-hydroxycoumarin), umbelliferone (7-hydroxycoumarin) and xanthyletin (6,7-dimethylpyranocoumarin) have also been detected in tissues from citrus inoculated with pathogens, such as *P. digitatum* and *Phytophthora* spp (Khan et al., 1985; Rodov et al., 1994; Afek et al., 1999).

It is also known that some primary metabolites are correlated with biotic and abiotic stress in diverse plant species (Khan et al., 2020). Previous research suggests that primary metabolism plays an important role during plant-pathogen interactions, for example, supporting cellular energy requirements for plant defense responses (Rojas et al., 2014). This phenomenon was also observed in the present study, in which ^1^H NMR-based metabolomics correlated sugars, amino acids, and organic acids with metabolic differences between the susceptible and resistant species*.* Besides, some of these compounds were also correlated with differences between controls and inoculated samples of the resistant species in which, along with flavonoids and coumarins, numerous primary metabolites increased after inoculation: choline, carbohydrates (fructose, glucose, and sucrose), amino acids (leucine, valine, and proline), and organic acids (fumaric acid and succinic acid).

Sugars are considered to be the major contributing factor in osmotic adjustment in most plant species. For example, the accumulation of soluble sugars is notably related to osmo-protection and scavenging of free radicals (Khan et al., 2018). Sugars are also involved in several biological processes, being structural cell constituents that act as a metabolic resource, which may contribute during stress. Moreover, they can act directly as negative signals or modifiers of the cell reactive pathways to induce stress response signals and increase plant resistance to environmental stress (Rosa et al., 2009; Ahmad et al., 2016; Khan et al., 2020).

Amino acids are also relevant primary metabolites in response mechanisms. They are precursors of proteins and other organic compounds such as nucleic acids that play an active role in counter reactions of plant against several stresses. For example, proline, plays crucial roles in the osmotic adjustment and protection of subcellular structures during abiotic and biotic stresses (Freitas et al., 2015). Organic acids have also been reported to be responsible for the increased plant response to abiotic stress, especially to drought tolerance (Khan et al., 2020) The role in this type of stress has been related to their participation in the energy production process, having been observed to increase or decrease in plants under severe stress conditions such as drought, as mentioned and osmotic stress (Sassi et al., 2010; Gao et al., 2012).

All these studies appear to confirm that primary metabolism is involved in defense related mechanisms. However, more work is needed to understand the direct correlation between the level of some primary metabolites in particular such as amino acids or organic acids. For example, proline might be involved in the defense mechanism of *C. latifolia* against *P. citricarpa*, since multivariate data analysis showed this amino acid and other primary metabolites to be correlated to the metabolic alteration in inoculated leaves of *C. latifolia* (resistant species). This is also the case for other fungal diseases such as, HLB, in which an observed accumulation of proline in *C. latifolia* was also observed in infected samples (Cevallos-Cevallos et al., 2011; Cevallos-Cevallos et al., 2012; Freitas et al., 2015). Nonetheless, there is no direct proof that the accumulation of proline is related to the defense responses of *C. latifolia*. Hence, the full clarification of its role requires further studies on the precursors and activities of the key enzymes in its metabolism (Freitas et al., 2015), including CBS.

In this study coumarins and furanocoumarins were identified in C. *latifolia*, which is resistant to *P. citricarpa*, the pathogen responsible for citrus black spot disease in many citrus species. However, when tested individually against the fungus, none showed significant activity, while the whole coumarin fraction isolated from the resistant species, containing all the individually tested compounds exhibited a mild inhibition with a MIC = 250 μg/ml, which might suggest a synergistic effect among the compounds present in the extract.

The bioactivity potentiating interaction between coumarins has been reported previously. For example, Ramírez-Pelayo et al. (2019) evaluated the activity of different ratios of combinations of 5,7-dimethoxycoumarin and 5-methoxypsoralen against *Colletotrichum* sp., all of which displayed significantly greater antifungal activity than the individual compounds. Furthermore, the authors reported that the fungistatic activity against *Colletotrichum* sp of this combination was greater than that of the phytoalexin scoporane, that is known for its strong toxicity against several pathogens.

The *in-silico* activity analysis of compound **1** revealed a high potential as a CDP-glycerol glycosyltransferase inhibitor, which is related to cell wall biosynthesis inhibitor activity. Moreover, compound **1** showed a significant potential to work against the enzyme NAD(P)^+^ -arginine ADP-ribosyltransferase. This enzyme is responsible for the transfer of the ADP-ribosyl group to target proteins, which results in its covalent modification (Maurer et al., 2011). However, the inhibition of this enzyme is considered a highly specific target for new antibacterials, since this biosynthetic pathway is limited to bacteria (Seibert et al., 2019). Compound **1** also showed a high potential for 2-dehydropantoate 2-reductase inhibition. This enzyme is responsible for pantothenate biosynthesis, a B complex vitamin which is the key precursor for the biosynthesis of coenzyme A. The inhibition of this pathway might be a potential target for antimicrobial agents (Leonardi & Jackowski, 2007). These activities support the fungicidal activity of this compound. Conversely, **5** showed a low potential for NAD(P)^+^ -arginine ADP-ribosyltransferase and 2-dehydropantoate 2-reductase inhibition. This could partially explain the absence of direct antifungal activity of this compound. However, it exhibits a higher potential as a membrane permeability enhancer than **1**. Since **5** also showed a moderate value for the inhibition of squalene epoxidase, its activity can be also related to the inhibition of the synthesis of cholesterol. This led to the hypothesis that compounds **5** and **1** could interact synergistically to potentiate the antifungal effects of **1**. This could also explain and support the activity of the coumarin fraction with lower MIC value. Similarly, compounds **2**, **3**, and **4**, which were present together with **1** and **5** in the coumarin fraction, also showed a high potential for CDP-glycerol glycosyltransferase inhibition. Furthermore, compounds **2** and **3** showed moderate activity as membrane permeability enhancers. All this indicates clearly that the antifungal potential of *C. latifolia* coumarins lies in their combination as confirmed by the lower MIC values of the coumarin rich fractions against *P. citricarpa* compared to that of individual coumarins.

Our conclusions coincide with that of other authors who have observed synergistic interactions among several types of coumarins as well as other types of metabolites in antimicrobial plant defence mechanisms (Hiruma, 2019). The hydroxycoumarin scopoletin, for example, showed a strong enhancement of its antifungal effect against *Fusarium verticillioides* (Saccardo) when combined with different compounds, such as vanillin, 4-hydroxy-3-methoxycinnamaldehyde, and pinoresinol (Carpinella et al., 2005).

This study performed with metabolomics analysis demonstrated a metabolic differentiation between leaves of *C. limon* and *C. latifolia*, which are susceptible and resistant to infection by *P. citricarpa*, respectively. The metabolomics analysis of the samples of *C. limon* and *C. latifolia*, revealed clear differences in their metabolome. These metabolic differences were mainly caused by phenolics, specifically coumarins. The presence of higher concentrations of this type of metabolites and of species-specific coumarins in *C. latifolia* were thus associated to its inherent resistance to *P. citricarpa*. The evaluation of the antifungal effect of five of these identified coumarins showed an inhibition of the fungus by combinations of these compounds. The higher activity of the coumarin-rich fraction suggested a potentiation of the activity of its components against *P. citricarpa*. This was also supported by the *in-silico* analysis of the chemical structures of the five coumarins. Thus, these results highlight the potential use of coumarin mixtures as potential biopesticides for the control of *P. citricarpa*.

## Data Availability

The original contributions presented in the study are included in the article/Supplementary Material, further inquiries can be directed to the corresponding author.
