# Supplementary material for: Metabolomic Investigation of Citrus latifolia and the Putative Role of Coumarins in Resistance to Black Spot Disease
Source: Front Mol Biosci. 2022 Jun 24;9:934401. doi: 10.3389/fmolb.2022.934401 (PMC9263546; doi:10.3389/fmolb.2022.934401)
Supplement: Supplementary file 1 [file Image5.PDF]

Supplementary Figures S 5A-F MS/MS spectra of annotated compounds in the coumarin fraction of *C. latifolia*, resistant to *Phyllosticta citricarpa*.

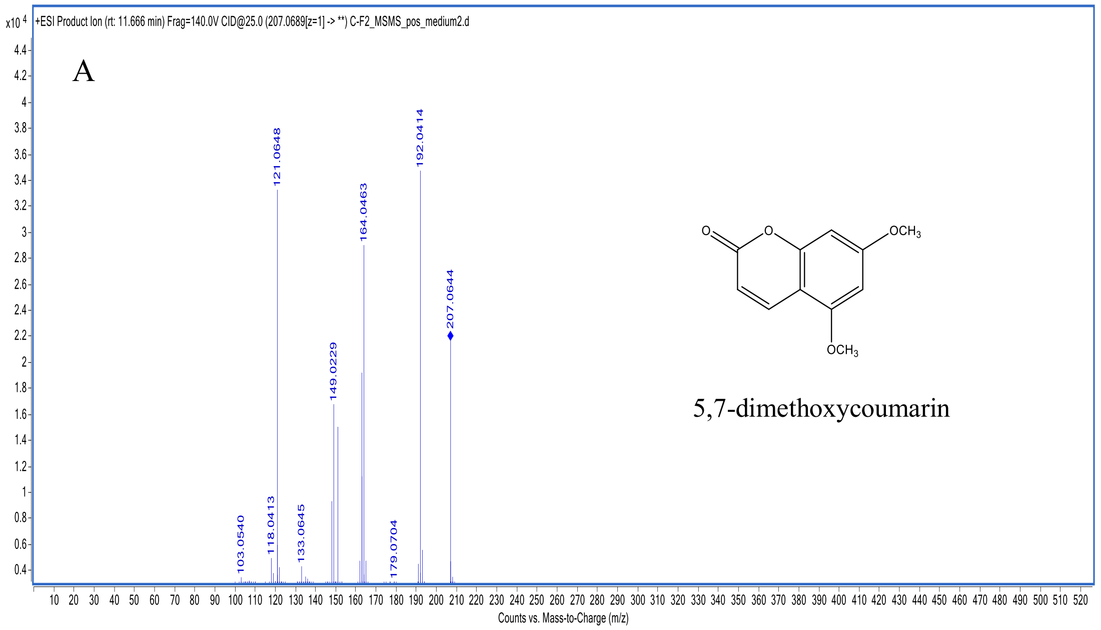

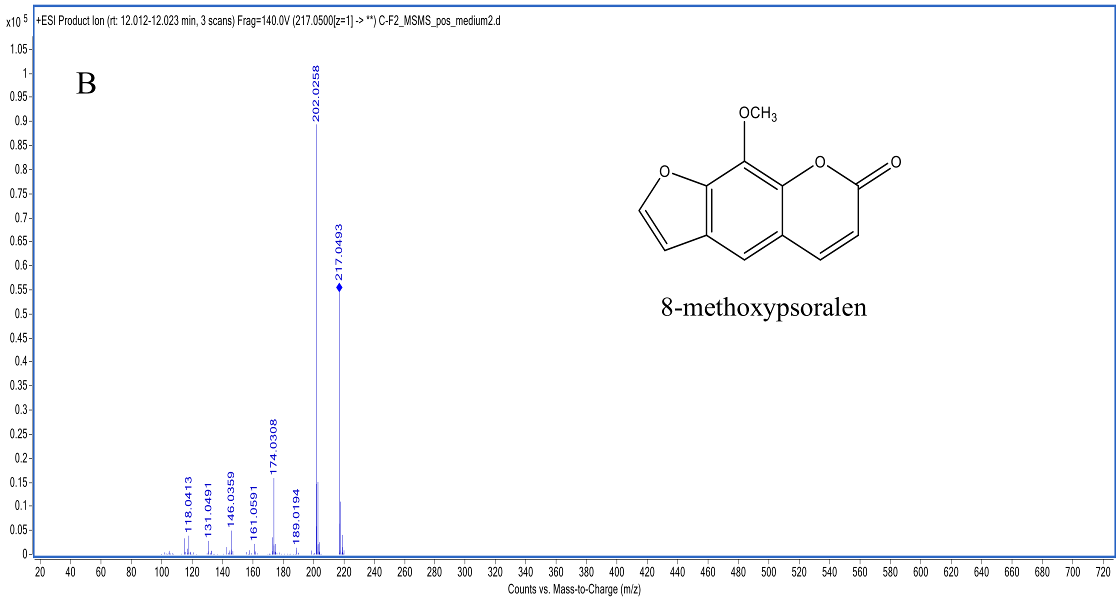

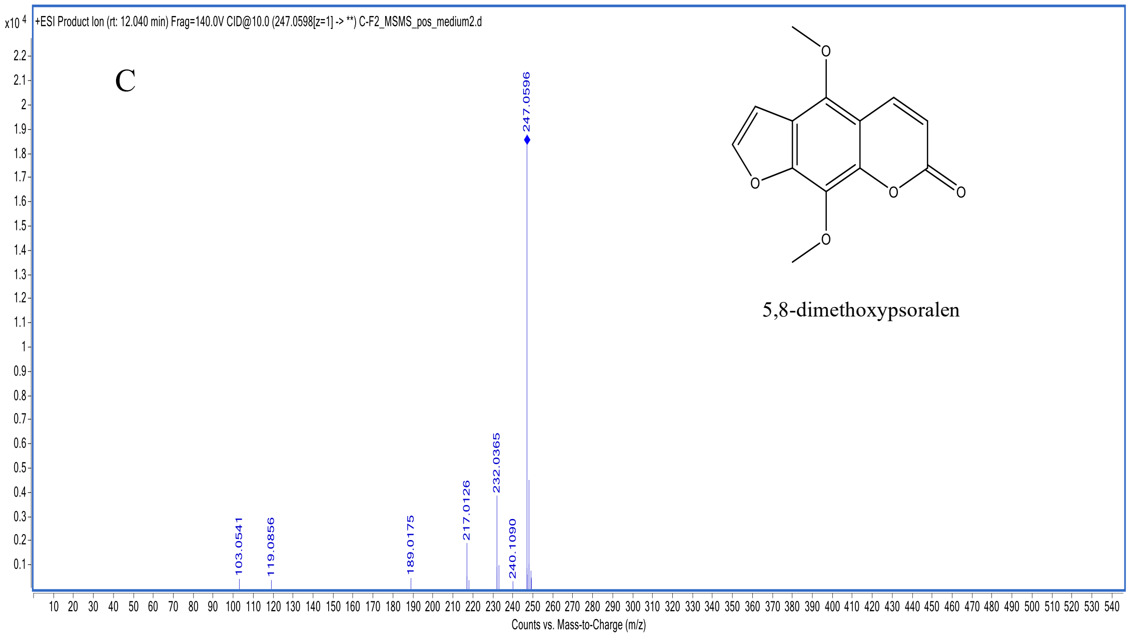
+ESI Product Ion (rt: 12.040 min) Frag=140.0V CID@10.0 (247.0598[z=1] -> **) C-F2_MSMS_pos_medium2.d
x10 4
C
247.0596
232.0365
217.0126
240.1090
189.0175
119.0856
103.0541
5,8-dimethoxypsoralen
Counts vs. Mass-to-Charge (m/z)

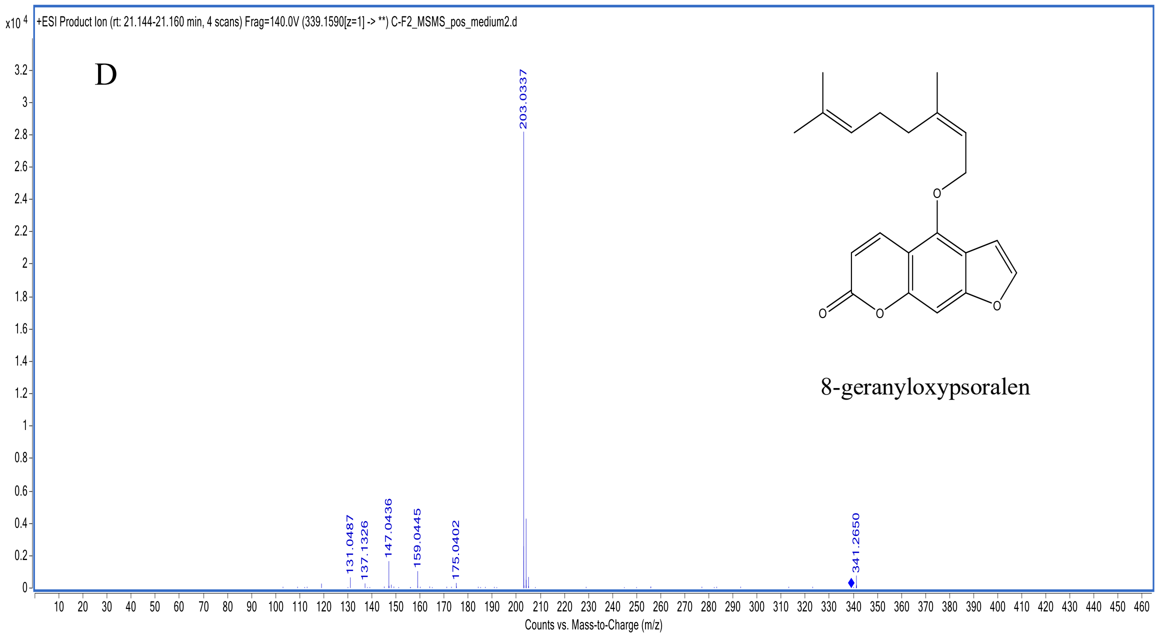
+ESI Product Ion (rt: 21.144-21.160 min, 4 scans) Frag=140.0V (339.1590[z=1] -> **) C-F2_MSMS_pos_medium2.d
x10 4
D
203.0337
147.0436
159.0445
131.0487
137.1326
175.0402
341.2650
8-geranyloxypsoralen
Counts vs. Mass-to-Charge (m/z)

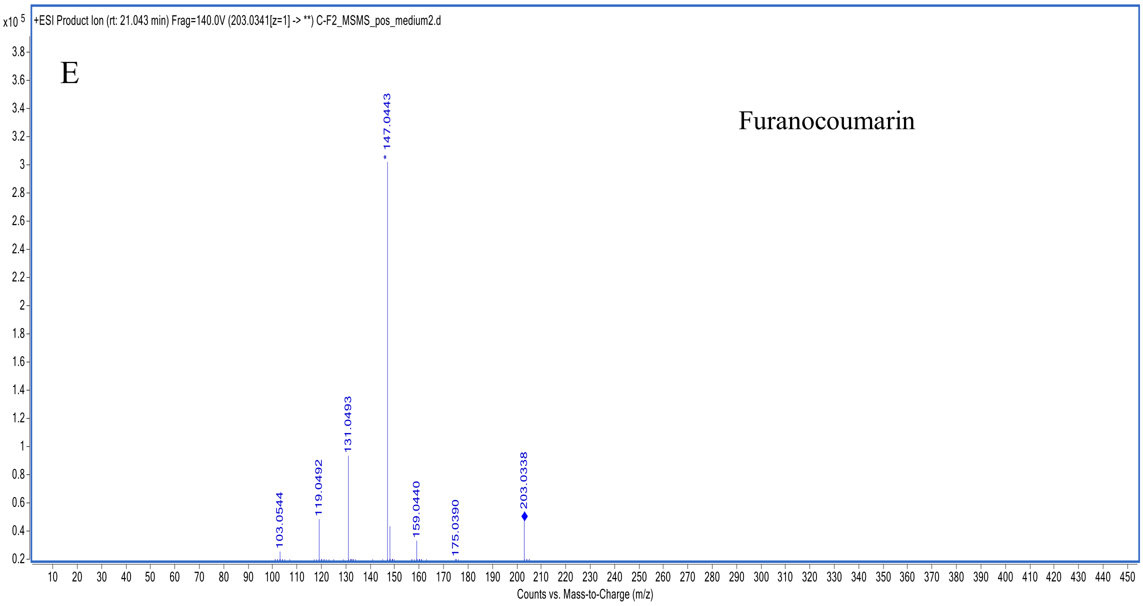
+ESI Product Ion (rt: 21.043 min) Frag=140.0V (203.0341[z=1] -> **) C-F2_MSMS_pos_medium2.d
E
Furanocoumarin
147.0443
131.0493
119.0492
103.0544
159.0440
175.0390
203.0338
Counts vs. Mass-to-Charge (m/z)

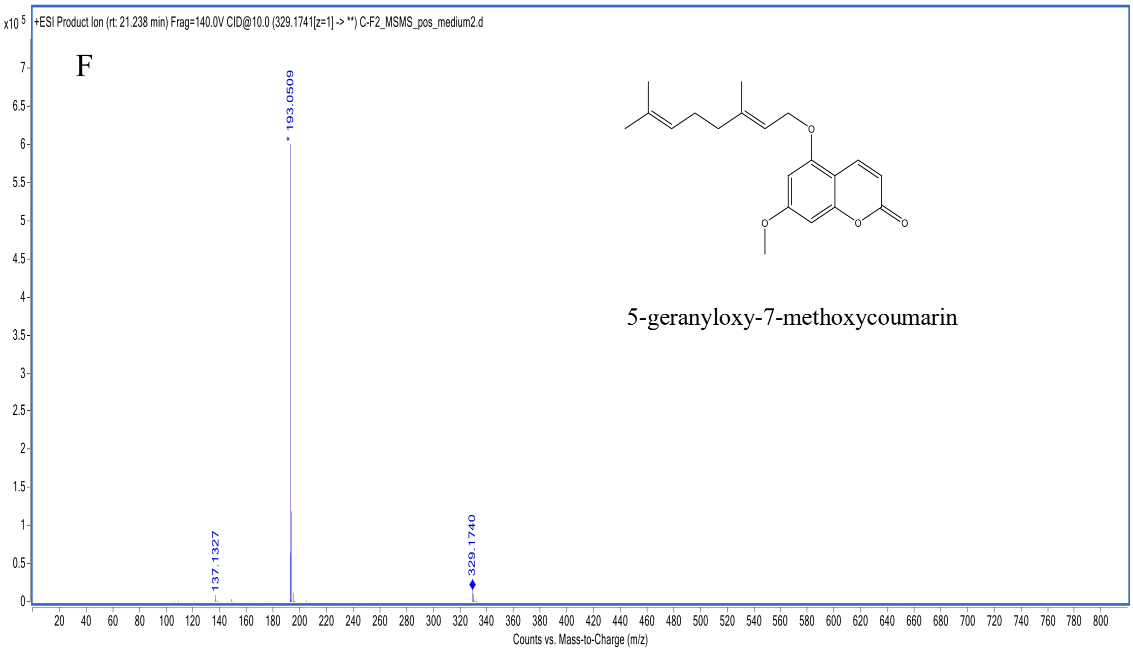
+ESI Product Ion (rt: 21.238 min) Frag=140.0V CID@10.0 (329.1741[z=1] -> **) C-F2_MSMS_pos_medium2.d
F
193.0509
137.1327
329.1740
5-geranyloxy-7-methoxycoumarin
Counts vs. Mass-to-Charge (m/z)
