# Supplementary material for: Metabolomic Investigation of Citrus latifolia and the Putative Role of Coumarins in Resistance to Black Spot Disease
Source: Front Mol Biosci. 2022 Jun 24;9:934401. doi: 10.3389/fmolb.2022.934401 (PMC9263546; doi:10.3389/fmolb.2022.934401)
Supplement: Supplementary file 2 [file Table1.DOCX]

Supplementary Table S-1| ^1^H NMR Chemical shift assignments of the compounds characterized in extracts of leaves citrus species.

| **Nº** | **Compound** | **Assignment** | **δ ^1^H** | **Multiplicity [J(Hz)]** | ***Species ^a^*** | |
| --- | --- | --- | --- | --- | --- | --- |
|  |  |  |  |  | ***C. latifolia*** | ***C. lemon*** |
| **Carbohydrates** | | | | | | |
| 1 | β-glucose | 1-CH | 4.54 | d (7,90) | D | D |
| 2 | α-glucose | 1-CH | 5.10 | d (3.87) | D | D |
| 3 | Sucrose | 1-CH | 5.38 | d (3.74) | D | D |
|  |  | 10-CH | 3.41 | m |  |  |
| 4 | Fructose | 1-CH | 4.36 | dd (1.9, 12.1) | D | D |
|  | **Organic Acids** | | | | | |
| 5 | Citric acid | 1-CH_2_ | 2.65 | d (9.46) | D | D |
|  |  | 2-CH_2_ | 2.68 | d (9.46) |  |  |
| 6 | Tartaric acid | H-2, H-3 | 4.30 | s | D | ND |
| 7 | Fumaric acid | H-2, H-3 | 6.65 | s | D | D |
|  |  |  |  |  |  |  |
| 8 | Succinic acid | H-2, H-3 | 2.52 | s | D | D |
| 9 | Formic acid | H-1 | 8.46 | s | D | D |
| 10 | Acetic acid | H-1 | 1.96 | s | D | D |
| 11 | Malonic acid | α-CH_2_ | 3.14 | s | D | D |
| 12 | α- Linolenic acid | α-CH_2_ | 0.95 | t (7.2) | D | D |
| 13 | γ-aminobutyric acid (gaba) | α-CH_2_ | 2.31 | m | D | D |
|  |  | γ-CH3 | 2.95 | t (7.24) | D | D |
|  | **Amino Acids** | | | | | |
| 14 | Alanine | β-CH_3_ | 1.45 | d (7.27) | D | D |
| 15 | Valine | α-CH | 3.68 | d (4.35) | D | D |
|  |  | β-CH | 2.31 | m |  |  |
|  |  | γ-CH_3_ | 0.94 | d (7.01) |  |  |
|  |  | γ`-CH_3_ | 1.15 | d (7.01) |  |  |
| 16 | Leucine | α-CH_2_ | 2.94 | dd (16.7, 3.96) | D | D |
|  |  | β-CH | 1.74 | m |  |  |
|  |  | γ-CH_3_ | 1.74 | m |  |  |
| 17 | Isoleucine | α-CH_2_ | 0.89 | t (7.13) | D | D |
|  |  | γ-CH_3_ | 3.21 | d (3.95) |  |  |
| 18 | Threonine | γ-CH_3_ | 1.31 | d (6.6) | D | D |
|  |  | β-CH | 4.11 | m |  |  |
| 19 | Tyrosine | H-2 | 7.19 | m | D | D |
|  |  | H-3 | 6.87 | m | D | D |
| 20 | Proline | α-CH | 4.01 | m | D | D |
|  |  | β-CH_2_ | 2.00-2.15 | m | D | D |
|  |  | γ-CH_2_ | 2.25-2.34 | m | D | D |
|  | **Other Compounds** | | | | | |
| 21 | Choline | CH_3_ | 3.20 | s | D | D |
| 22 | Coumarins | H-3  H-4 | 6.23, 6.29  8.07 | d (9.6)  d (9.6) | D | D |

D, detected; ND, non-detected; m, multiplet; dd, double doublet; t, triplet; d, doublet; s, singlet.
