# Supplementary material for: Metabolomic Investigation of Citrus latifolia and the Putative Role of Coumarins in Resistance to Black Spot Disease
Source: Front Mol Biosci. 2022 Jun 24;9:934401. doi: 10.3389/fmolb.2022.934401 (PMC9263546; doi:10.3389/fmolb.2022.934401)
Supplement: Supplementary file 3 [file Image4.PDF]

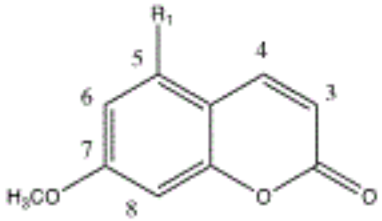

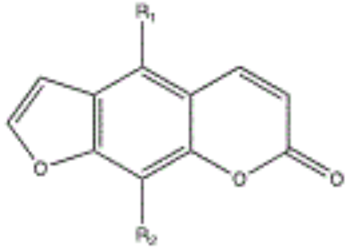

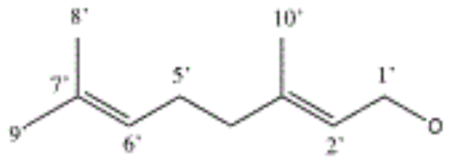

geranyloxy

1, R_1 = OCH_3
5, R_1 = geranyloxy

2, R_1 = H, R_2 = OCH_3
3, R_1 = OCH_3, R_2 = OCH_3
4, R_1 = geranyloxy, R_2 = H

Supplementary Figure S4| Compounds isolated and identified from *Citrus latifolia* (resistant species) and tested against *Phyllosticta citricarpa*; **1:** 5,7-dimethoxycoumarin; **2**: 8-methoxypsoralen; **3**: 5,8-dimethoxypsoralen; **4**: 8-geranyloxypsoralen; **5**: 5-geranyloxy-7-methoxycoumarin.
