# Supplementary material for: Metabolomic Investigation of Citrus latifolia and the Putative Role of Coumarins in Resistance to Black Spot Disease
Source: Front Mol Biosci. 2022 Jun 24;9:934401. doi: 10.3389/fmolb.2022.934401 (PMC9263546; doi:10.3389/fmolb.2022.934401)
Supplement: Supplementary file 4 [file Image2.PDF]

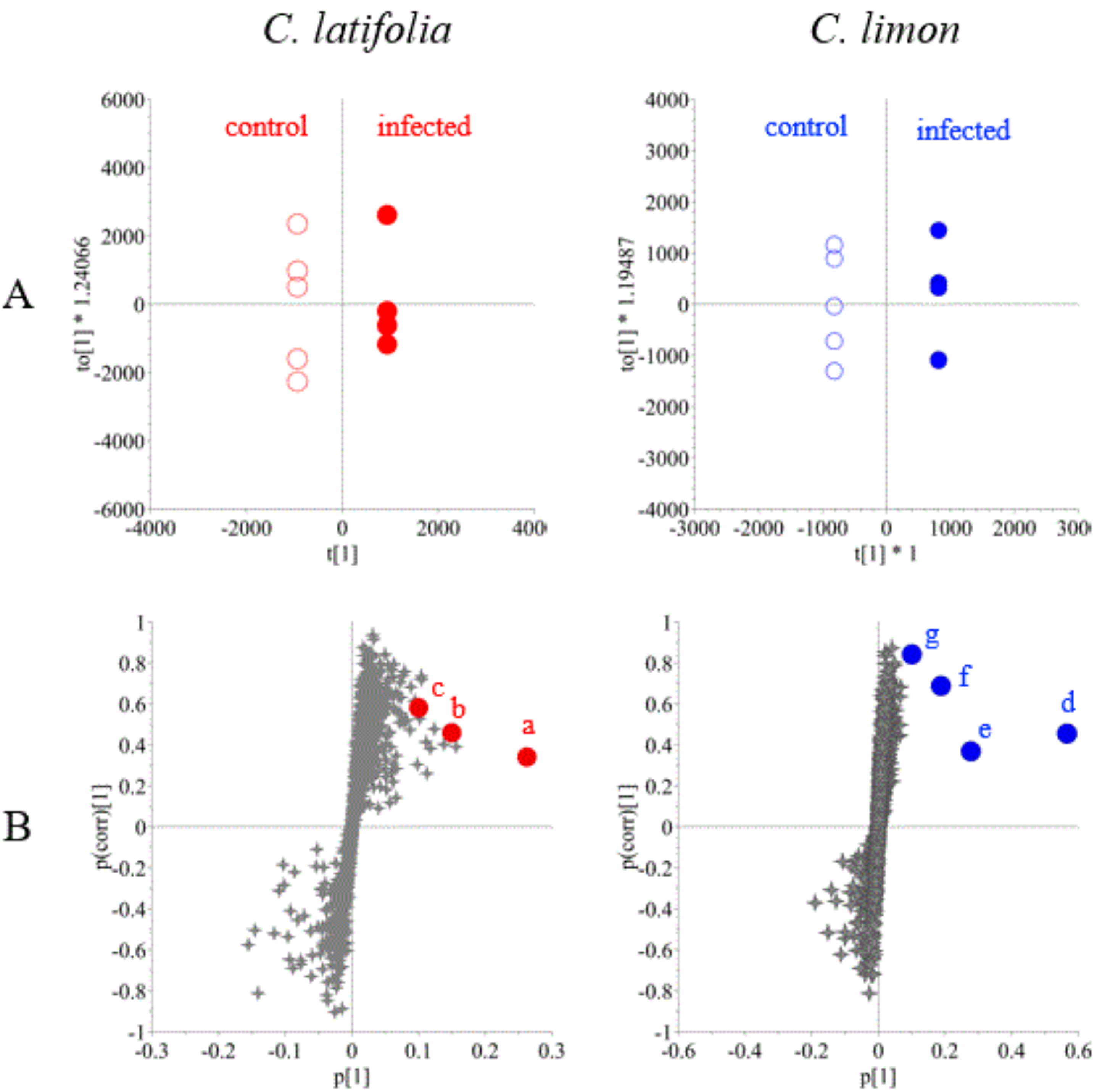

Supplementary Figure S2| (A) OPLS-DA and (B) Loading scores of *C. lemon* (S) x *C. latifolia* (R), based on LC-MS/MS 60 days analysis.
