# Supplementary material for: Metabolomic Investigation of Citrus latifolia and the Putative Role of Coumarins in Resistance to Black Spot Disease
Source: Front Mol Biosci. 2022 Jun 24;9:934401. doi: 10.3389/fmolb.2022.934401 (PMC9263546; doi:10.3389/fmolb.2022.934401)
Supplement: Supplementary file 5 [file Image3.PDF]

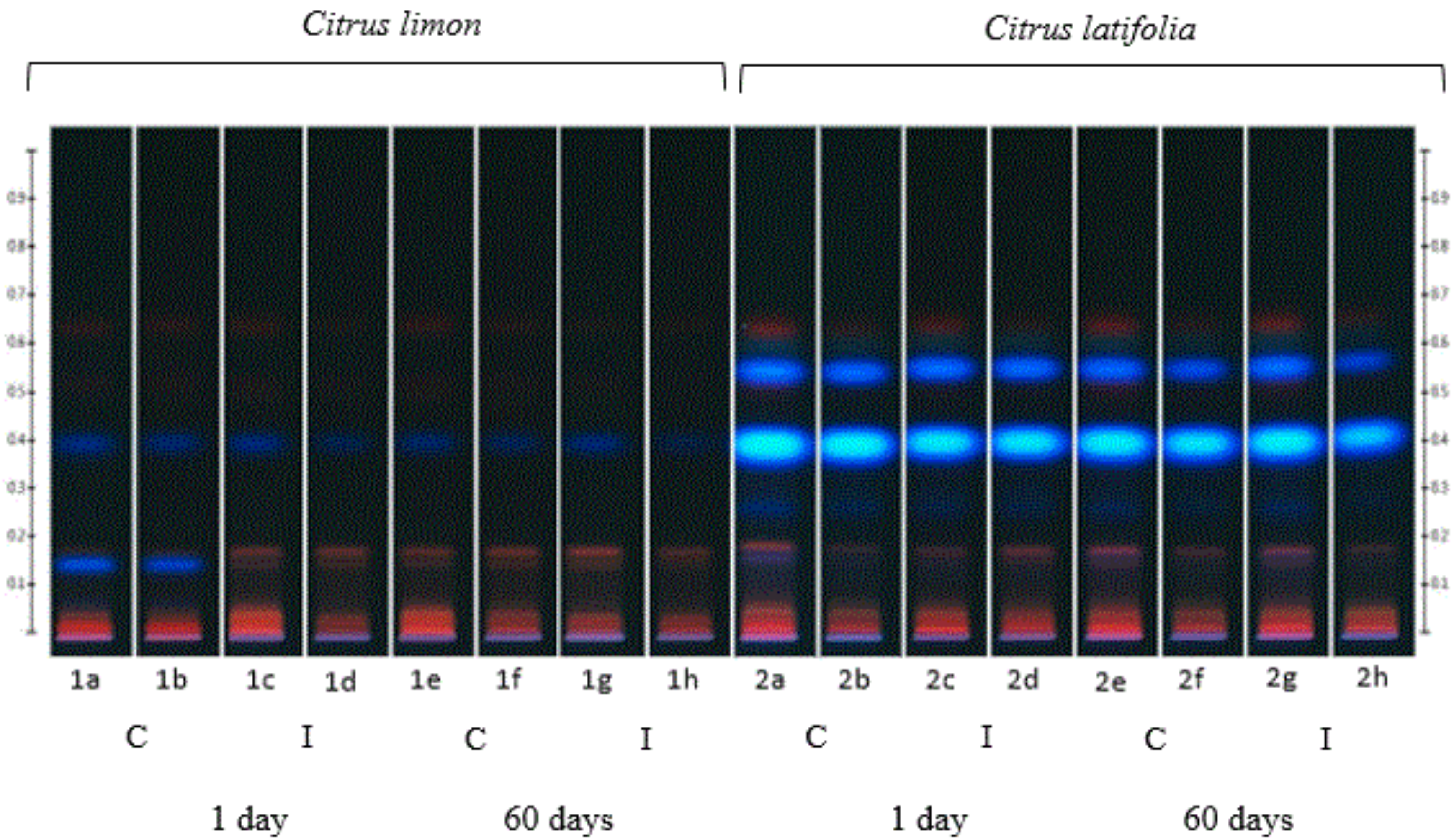

Supplementary Figure S3| High Performance Thin Layer Chromatography (HPTLC) analysis of *C. lemon* and *C. latifolia* samples. Non-polar metabolites eluted in toluene-ethyl acetate (8:2, v/v) without derivatization. Plates are visualized at 366 nm. Species/ control and inoculated samples; 1a/1b: *C. limon* control 1 day, 1c/1d: *C. limon* inoculated 1 day, 1e/1f: *C. limon* control 60 days, 1g/1h: *C. limon* inoculated 60 days, 2a/2b: *C. latifolia* control 1 day, 2c/2d: *C. latifolia* inoculated 1 day, 2e/2f: *C. latifolia* control 60 days, 2g/2h: *C. latifolia* inoculated 60 days.
