# Supplementary material for: Metabolomic Investigation of Citrus latifolia and the Putative Role of Coumarins in Resistance to Black Spot Disease
Source: Front Mol Biosci. 2022 Jun 24;9:934401. doi: 10.3389/fmolb.2022.934401 (PMC9263546; doi:10.3389/fmolb.2022.934401)
Supplement: Supplementary file 6 [file DataSheet1.pdf]

Supplementary Figures S6 A-M| 1D and 2D NMR spectra of 5,7-dimethoxycoumarin isolated from *Citrus latifolia*.

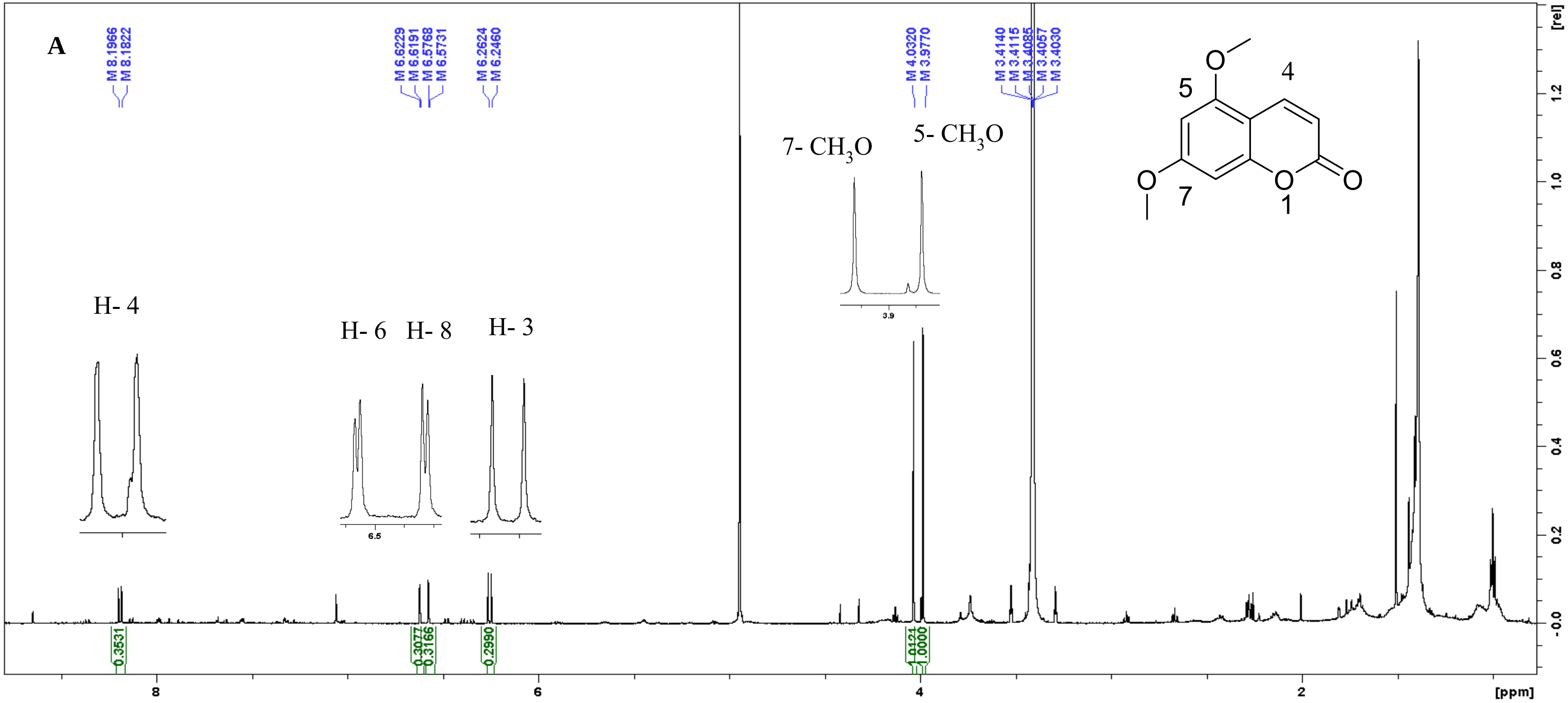

^{1}H NMR spectra of compound **01** (600 MHz in CH_3OH-d4).

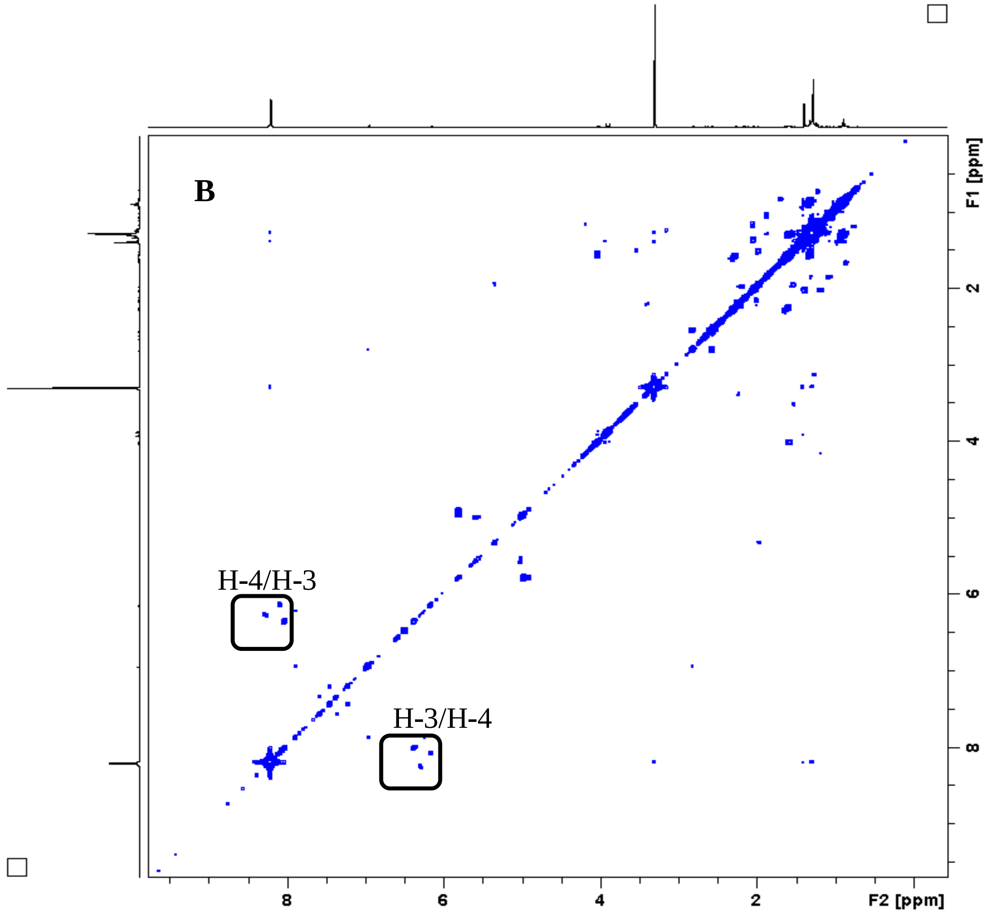

^{1}H COSY spectra of compound **01** (600 MHz in CH_3OH-d4)

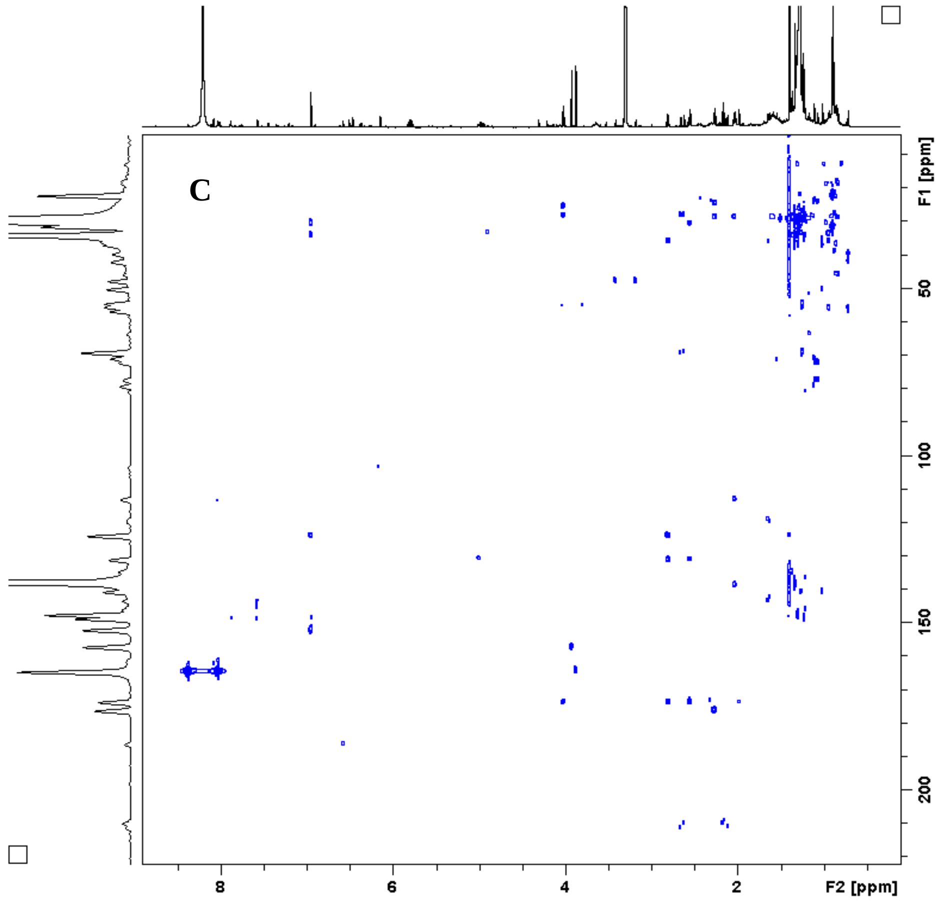

^{1}H, ^{13}C HMBC spectra of compound **01** (600 MHz in CH_3OH-d4)

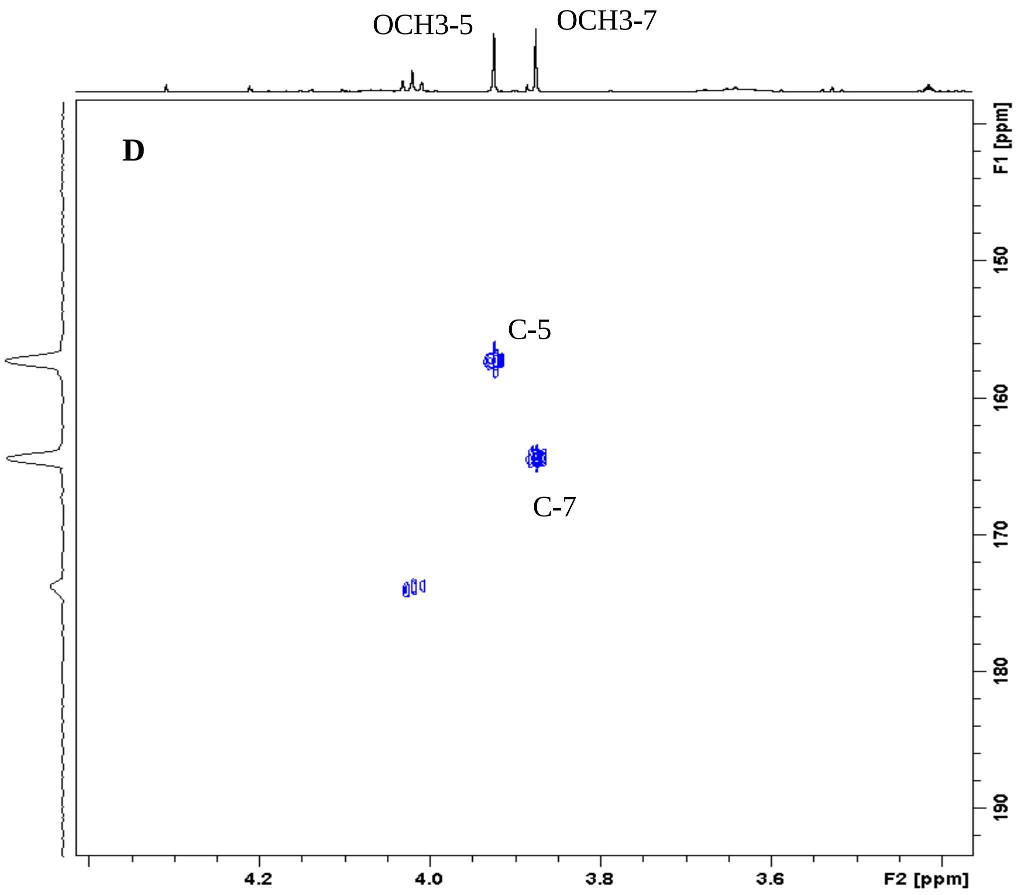

^1H, ^{13}C HMBC spectra of compound **01 (**600 MHz in CH_3OH-d4)

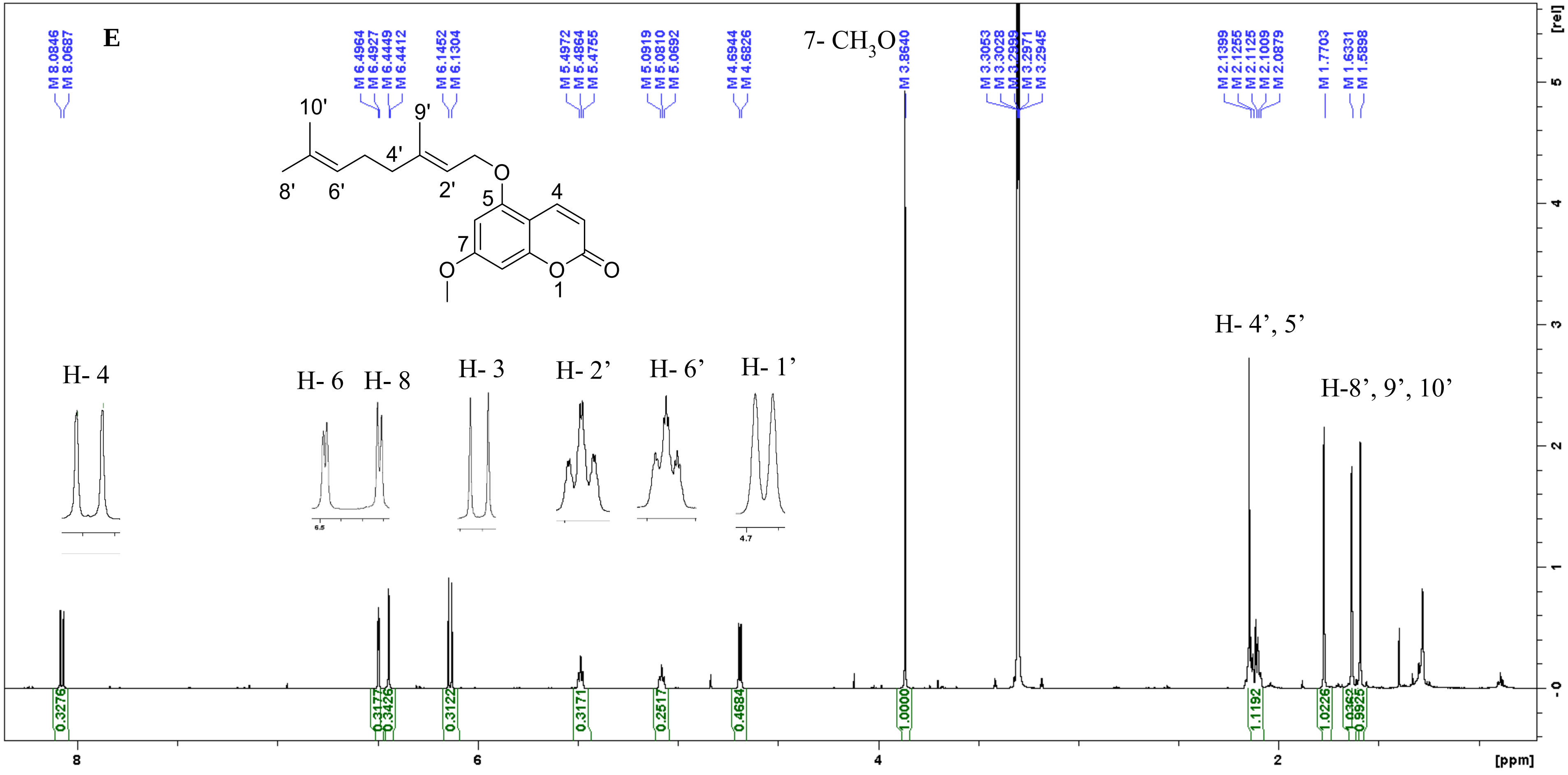

^{1}H NMR spectra of compound **02** (600 MHz in CH_3OH-d4).

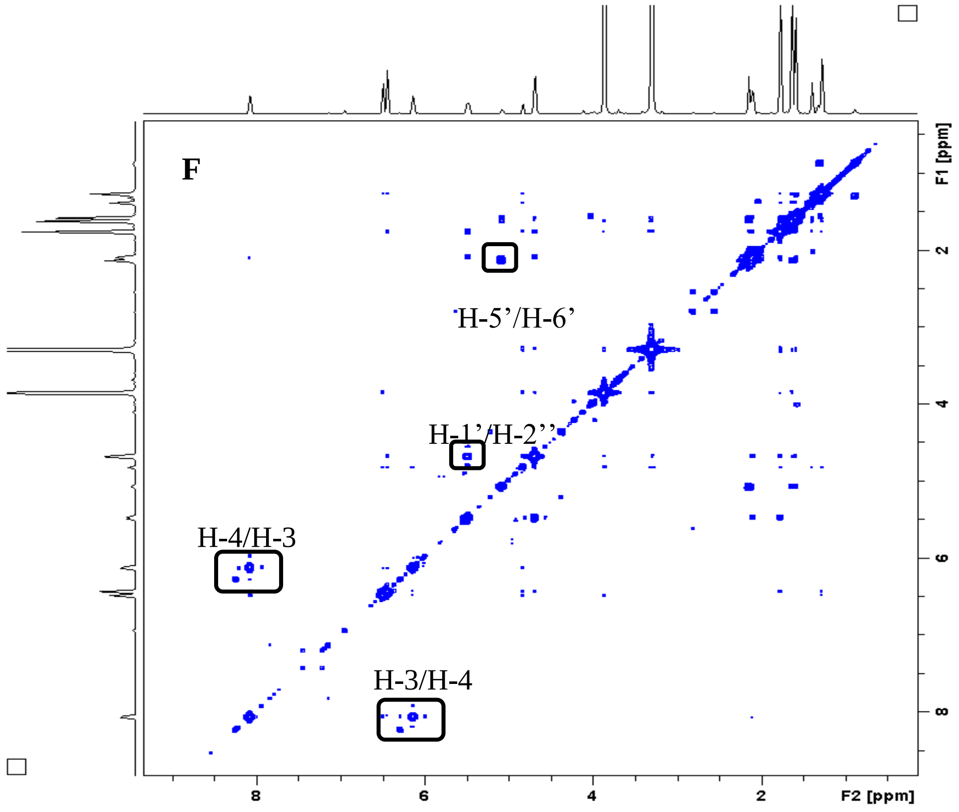

^{1}H COSY spectra of compound **02 (**600 MHz in CH_3OH-d4).

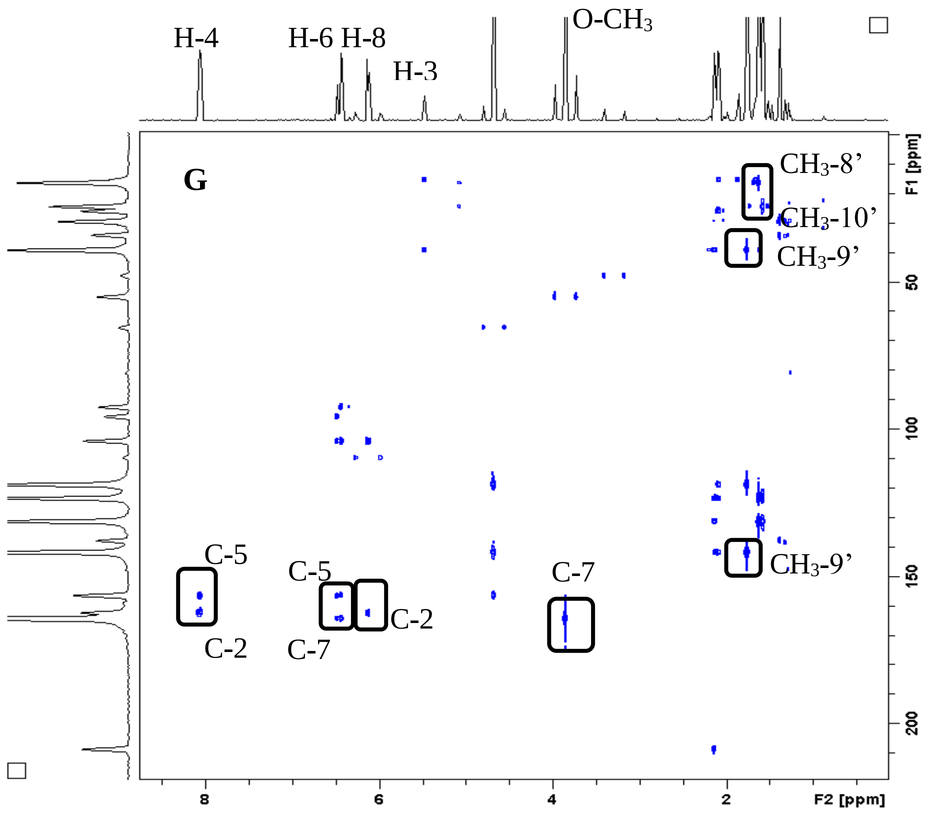

^{1}H, ^{13}C HMBC spectra of compound **02** (600 MHz in CH_3OH-d4).

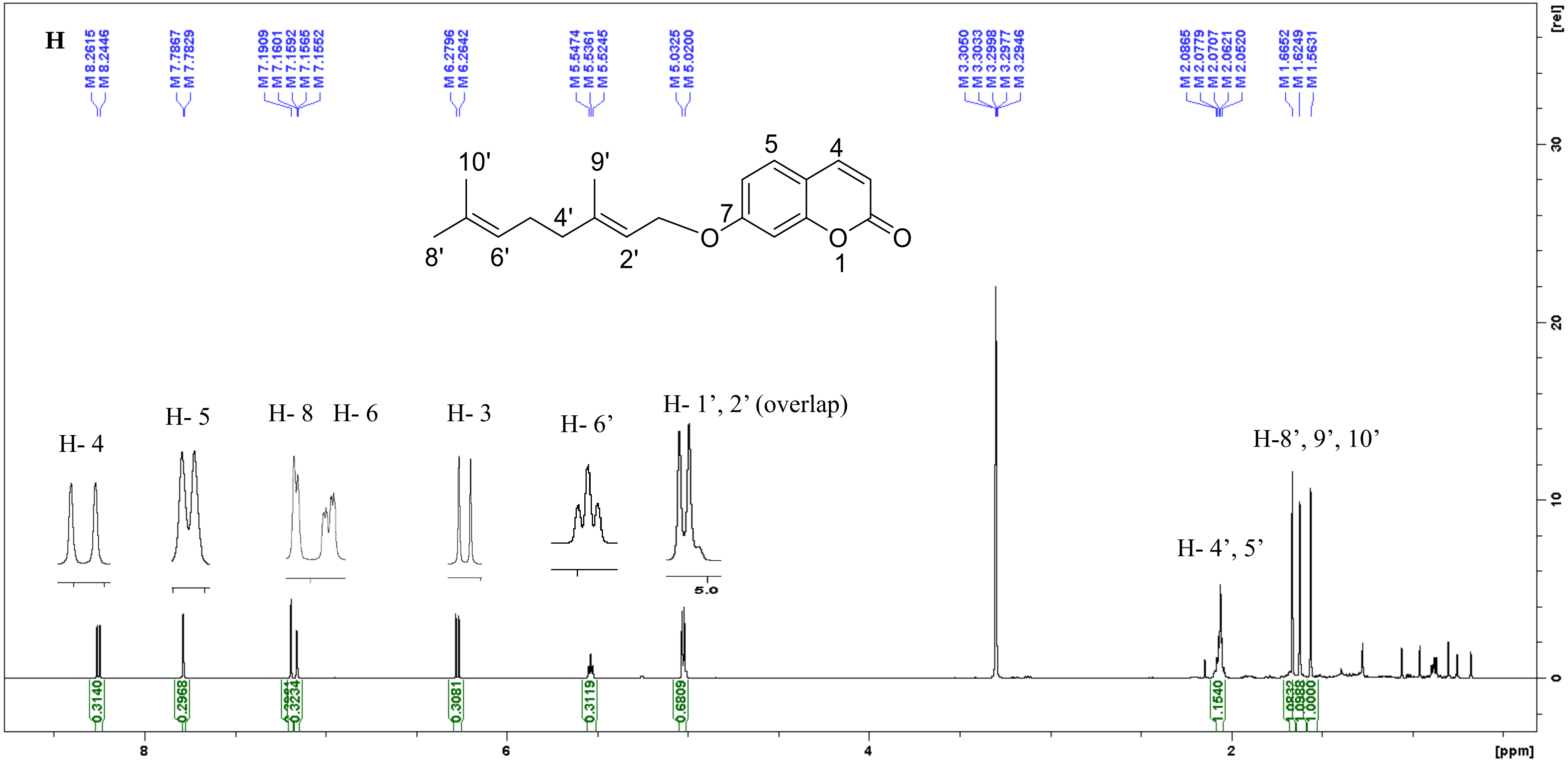

^{1}H NMR spectra of compound **03** (600 MHz in CH_3OH-d4).

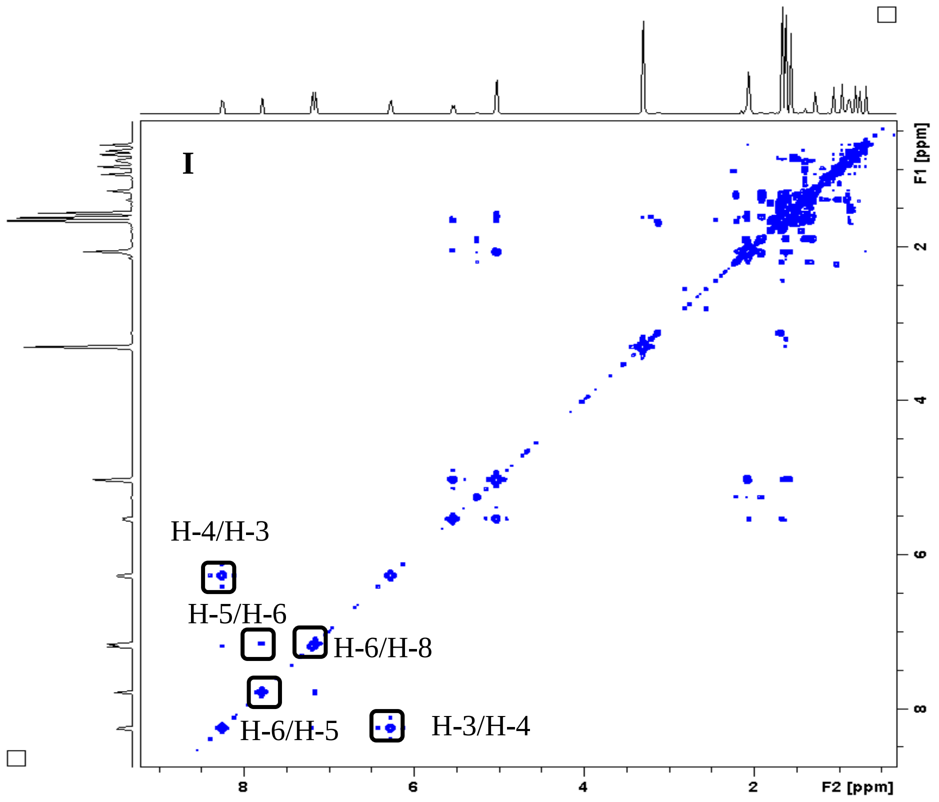

^{1}H COSY spectra of compound **03 (**600 MHz in CH_3OH-d4).

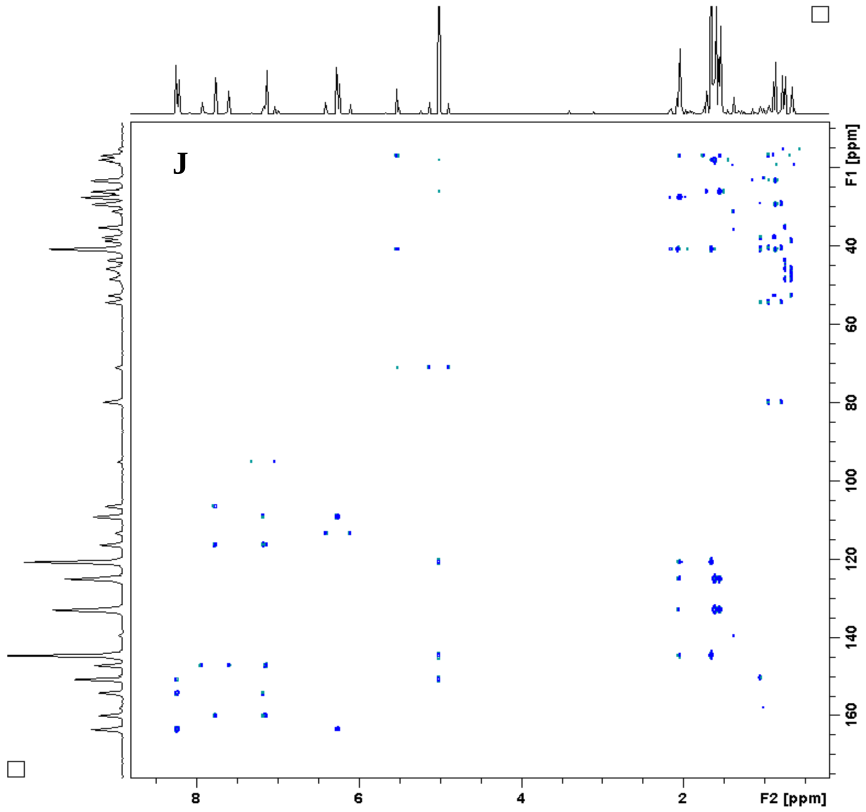

^{1}H, ^{13}C HMBC spectra of compound **03** (600 MHz in CH_3OH-d4).

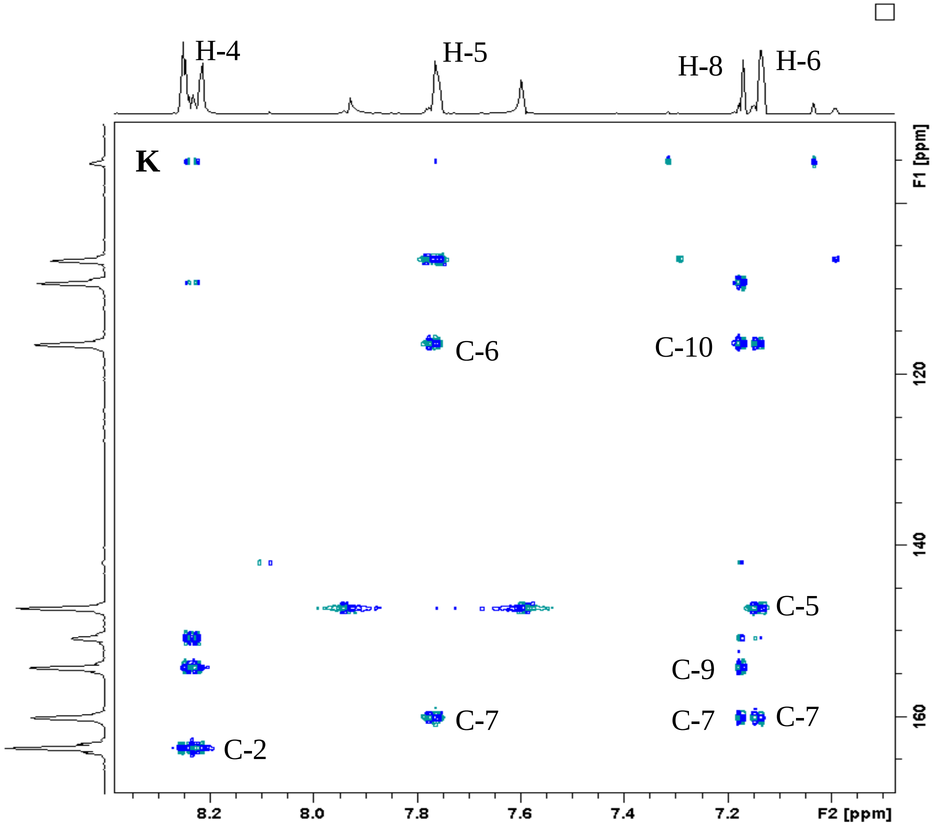

^{1}H, ^{13}C HMBC spectra of compound **03** (600 MHz in CH_3OH-d4).

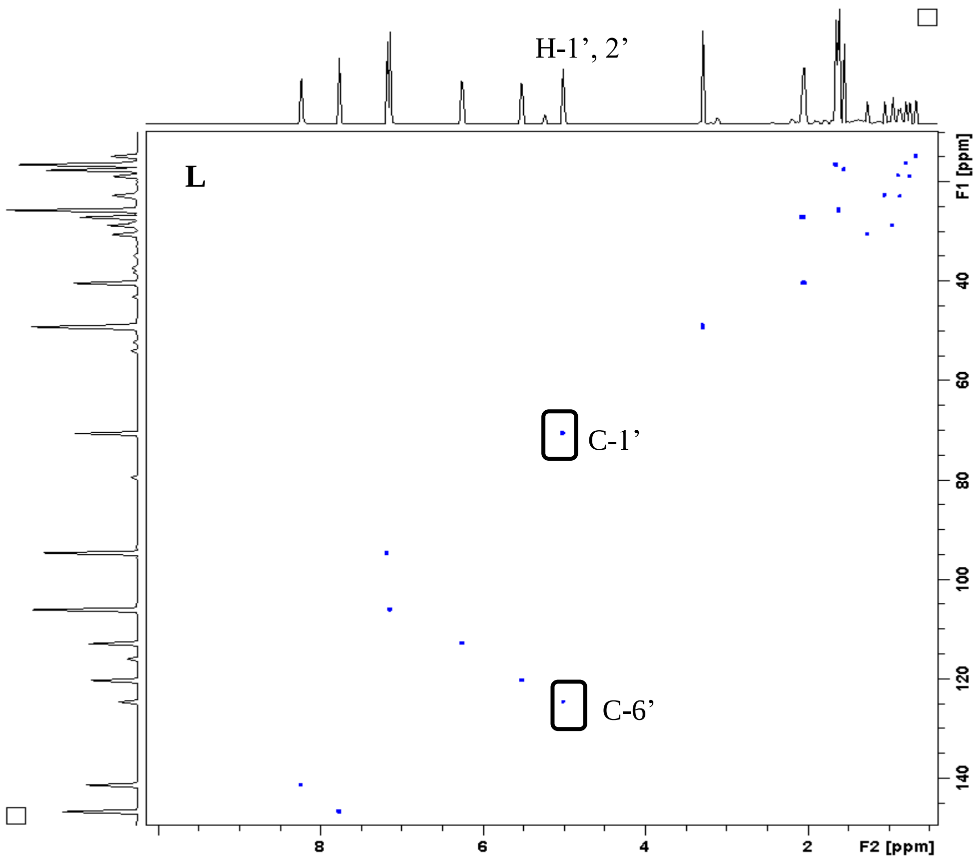

^{1}H, ^{13}C HSQC spectra of compound **03** (600 MHz in CH_3OH)

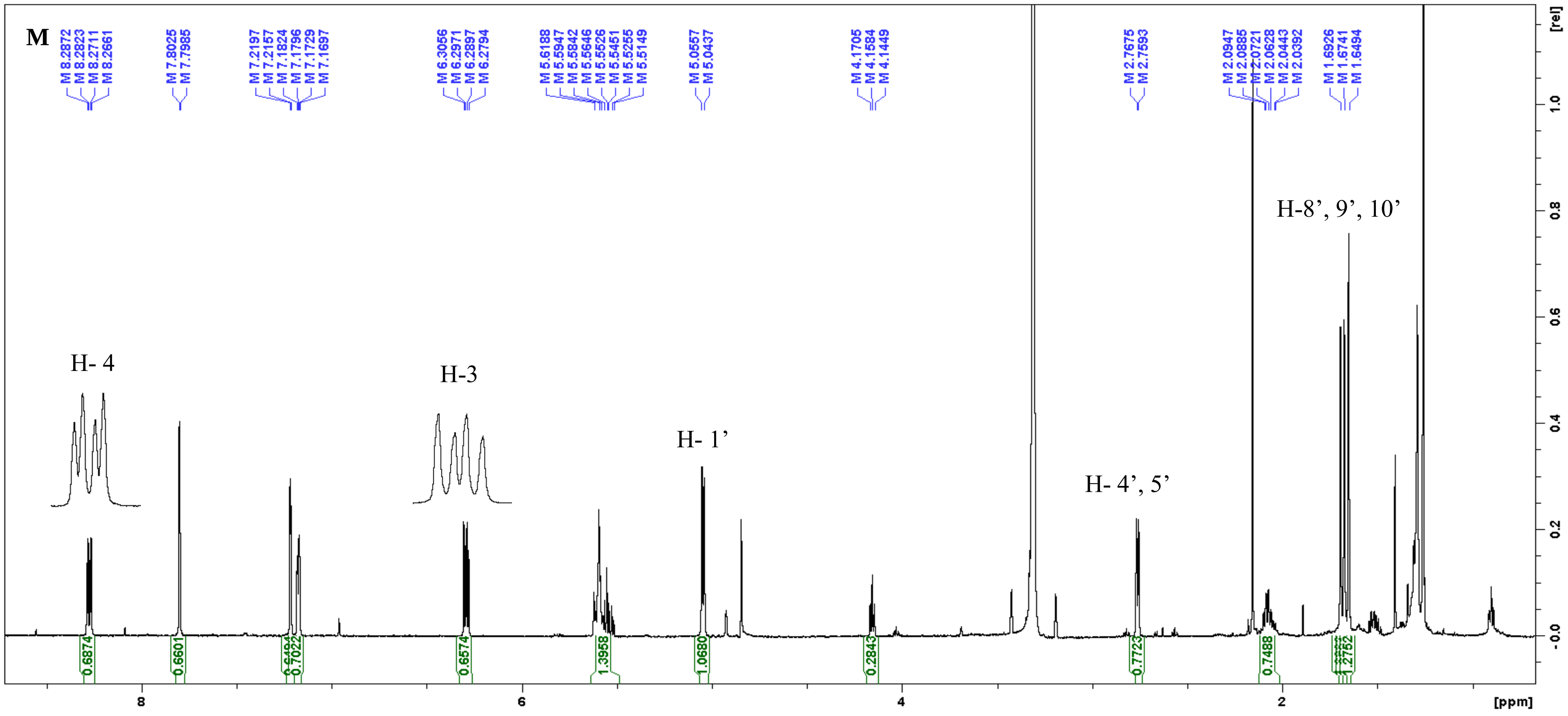

^{1}H NMR spectra of compound **04 (**600 MHz in CH_3OH-d4).
